# Advances in Image-Based Diagnosis of Diabetic Foot Ulcers Using Deep Learning and Machine Learning: A Systematic Review

**DOI:** 10.3390/biomedicines13122928

**Published:** 2025-11-28

**Authors:** Haifa F. Alhasson, Shuaa S. Alharbi

**Affiliations:** Department of Information Technology, College of Computer, Qassim University, Buraydah 52571, Saudi Arabia; shuaa.s.alharbi@qu.edu.sa

**Keywords:** diabetes mellitus, diabetic foot ulcers, dfu dataset, machine learning, deep learning, convolutional neural networks, thermogram

## Abstract

**Background/Objectives:** This review systematically assesses machine learning (ML) and deep learning (DL) applications using images to diagnose diabetic foot ulcers (DFUs), focusing on detection, segmentation, and classification. The study explores trends, challenges, and quality measurements of the reviewed research. **Methods:** A comprehensive search was conducted in October 2025 across 14 databases, covering studies published between 2010 and 2025. Studies employing ML/DL for DFU diagnosis with accurate measurements were included, while those without image-based methods, AI techniques, or relevant outcomes were excluded. Out of 4653 articles initially identified, 1016 underwent detailed review, and 102 met the inclusion criteria. **Results:** The analysis revealed that ML/DL models are effective tools for DFU diagnosis, achieving accuracy between 0.88 and 0.97, specificity between 0.85 and 0.95, and sensitivity between 0.89 and 0.95. Common methods included Support Vector Machines (SVMs) for ML and U-Net or fully convolutional neural networks (FCNNs) for DL. Recent studies also explored thermal infrared imaging as a promising diagnostic technique. However, only 45% of segmentation datasets and 67.3% of classification datasets were publicly accessible, limiting reproducibility and further development. **Conclusions:** This review provides valuable insights into trends and key findings in ML/DL applications for DFU diagnosis. It highlights the need for improved data availability and sharing to enhance reproducibility, accuracy, and reliability, ultimately improving patient care.

## 1. Introduction

Diabetic foot ulcers (DFUs) are the most common complication of Diabetes Mellitus (DM) that typically do not heal and, as a result, often result in amputation of the lower extremity. Epidemiologically, the number of DM patients around the globe is expected to grow from approximately 589 million adults in 2025 to 850 million in 2050, resulting in one of the most widespread chronic conditions in the world [1]. A DFU will occur in every third of patients with diabetes through their lifetime [2,3,4]. The economic impact of complications of diabetes is immense as well. Diabetes-related healthcare costs are projected to surpass USD 760 billion per year by 2025, with USD 45.9 billion exclusively targeted for diabetic neuropathy care in the USA [1].

The world market for advanced wound care is calculated to reach 22 billion by 2024 [5]. In the UK, DFU treatment alone costs an estimated GBP 580 million per year, as well as expenditures in other primary care settings [6]. Foot complications are a major contributor to medical expenses, with foot disease accounting for 50% of all diabetes admissions to hospitals [7]. Jodheea et al. [8] described in detail how geographical health economics, their effect on healing, as well as the factors that affect financial costs of DFUs, especially after the COVID-19 epidemic.

Early effective management of DFUs including education, blood sugar control, wound debridement, advanced dressing, offloading, advanced therapies, and in some cases surgery can reduce the severity of complications. Effective ulcer management is limited by several factors: (i) the results that are used to guide management processes are usually slow to reach clinical settings, (ii) the progression of DFU severity is hard to distinguish visually, and (iii) there are many DFU classification systems considering the proportion and types of tissue that are visually distinguishable [9,10,11,12]. DFU diagnosis is currently delivered by a specialist through visual examination. The complexity of providing the diagnosis can make this method time-consuming and lead to misdiagnoses. Currently, the accepted practice involves a trained clinician testing a patient’s feet manually with a hand-held nylon mono-filament probe. The procedure is time-consuming, labor-intensive, requires special training, is prone to error, and exhibits poor reproducibilityb [13]. The widespread uptake and acceptance of wearable and digital health technologies provides a means to monitor major risk factors associated with DFUs in a timely way [14]. This empowers patients in self-care and allows effective delivery of remote monitoring and multi-disciplinary prevention needed for those at-risk people. Technologies developed to enhance ulcer diagnostics and care plans have the potential to revolutionize diabetic foot care. Najafi et al. [15] summarize some of the promising developments in the area of digital health that may promote the prevention and management of DFUs. For the classification of diabetic foot ulcers, ref. [16] compares hybrid convolutional neural network models. The purpose of this comparison is to optimize deep learning approaches in clinical settings so that better diagnosis and treatment can be planned.

DFUs are considered a leading cause of hospitalization in patients with diabetes [17,18] and they can progress to severe infection, gangrene, amputation, and even death in the absence of proper care [19]. There is an urgent demand for reliable and quick management in diabetic patients, and Machine Learning (ML) and Deep Learning (DL) are great options to improve healthcare systems [20] through the prediction of future diabetes progression. ML/DL algorithms in DFU healthcare are becoming an increasingly popular approach [21]. ML algorithms are characterized by their ability to learn and adapt over time from raw data without being explicitly programmed [11], which has created a lot of excitement in the research community.

It is a complex task to find a suitable algorithm for the real-world circumstances of the DFU diagnostic process. Challenges include missing the early stages of ulceration, low quality of images available for DFU documentation (noise, shadow, blurring etc.), false positive detection cases (malformed toenails, deep wounds, folded amputation scars), fresh inflammation, and the variation of curved wound size, all of which can result in unnecessarily complicated treatments for both the patient and the healthcare system.

Over the years, several researchers studied to create mechanized systems for automatic DFU detection with the aid of ML with the objective to make it fast and accurate. Classical ML algorithms have emerged as a potential solution for automating DFU detection in the field of AI. In general, ML uses previous experience to improve the given results [22]. DL algorithms are a subset of ML algorithms that typically involve learning representations at different hierarchy levels to enable building complex concepts out of simpler ones. DL can be classified as supervised, unsupervised, and reinforcement learning. These ideas and efforts can be classified into two approaches: one uses the same classical method in which the first step is the preprocessing step, followed by segmentation, feature extraction, and then the classification step, while the second uses direct classification after the preprocessing step. Whether the approaches concentrate on segmentation, feature extraction, or classification, the tendency is to use the benefits of ML/DL techniques to obtain more accurate results. The various applications of ML/DL in the DFU field include: improving clinical decision-making based on ulcer classification, data analysis for risk and automated classification, or as an application for mobile devices for segmentation and classification.

### Purpose and Motivation

This study rationally focuses on reviewing the current state of Artificial Intelligence (AI) in the DFU field including detection, segmentation, and classification. The purpose of this study is to summarise the ML/DL methods used to improve DFU care in healthcare settings. It is hypothesized that ML models can assist specialists in achieving more accurate diagnoses compared with individual DFU specialists. The motivations for the project are:

- To engage in the efforts to find viable solutions for diabetic foot detection, thus assisting the DFU specialists in diagnosis and treatment.
- Analyze new trends of approaches used in the automatic DFU detection field. Thus, this paper focuses on presenting growth trends in the use of ML/DL techniques.

The detection and recognition of DFUs have been the subject of substantial research involving computer vision methods. However, systematic comparisons of deep learning and machine learning based on object detection frameworks are lacking [23]. There are many ML/DL models to explore and investigate throughout the literature for each target technique. The main technical gap is to identify the most suitable model architecture for a specific type of image dataset such as colored and thermal images. From this gap, the first question is formulated. In order to compare these models, the performance of these models is validated with different types of validation metrics. The second research question can be derived from this issue. However, a vast selection of datasets are available for model training, but there is no indication of which characteristics of an image dataset are most beneficial for the training process. This gap helps to develop the third research question.

This paper aims to systematically review recent advancements in ML/DL models for detecting, classifying, and segmenting diabetic foot ulcers (DFUs). The focus is on trends, challenges, and the quality of evidence reported in the literature. The study also highlights gaps in dataset availability and evaluation metrics, providing key insights to guide future research.

## 2. Materials and Methods

Section 2 presents the search strategy and criteria used to select relevant studies on ML/DL techniques for DFU diagnosis. The focus is on identifying trends, evaluating dataset usage, and comparing performance metrics. This systematic review has been registered with INPLASY (International Platform of Registered Systematic Review and Meta-analysis Protocols) under the registration number INPLASY2022110128.

### 2.1. Research Questions

- Research Question 1 (RQ1): What is the most effective ML/DL model for providing an optimal diagnosis for DFUs?
- Research Question 2 (RQ2): How should the performance of the models be compared using the optimal set of validation metrics?
- Research Question 3 (RQ3): What are the characteristics of the DFU image database that are required for the training of the diagnostic model?

### 2.2. Inclusion and Exclusion Criteria

Articles were selected based on the following criteria:

- **Inclusion Criteria:**
  – Articles must focus on ML/DL applications related to DFU detection, classification, or segmentation.
  – Articles must include references to or the creation of datasets used for model assessment.
  – Articles must provide a full-text version and include accuracy measurements for the results.
- **Exclusion Criteria:**
  – Preprinted articles or those without peer review were excluded [24].
  – Articles without images or those lacking AI applications were excluded [25,26,27,28].
  – Articles focusing on tasks outside detection, classification, or segmentation (e.g., improving image quality for downstream tasks [29]) were excluded.
  – Articles without accuracy measurements for the reported results were excluded.

This criteria reduced the number of eligible articles to 102. All selected articles were fully reviewed, and our analysis of AI trends in DFU diagnosis over the years is based on these developments. This review adheres to the PRISMA guidelines [30]—see Supplementary File S1—for Preferred Reporting Items for Systematic reviews and Meta-Analyses of diagnostic test accuracy studies. It is limited to image-based machine learning and deep learning models for diabetic foot ulcer diagnosis. Aligning this aim, we included studies that reported performance metrics, including accuracy, sensitivity, specificity, or related diagnostic measures for detection, classification, or segmentation tasks. Studies focusing on other aspects, such as usability, implementation, or decision-analytic metrics, were excluded. These elements are important for clinical adoption, but are beyond the scope of this review, which aims to assess image-based models from a technical perspective.

### 2.3. Resources Selection

**Figure 1:**
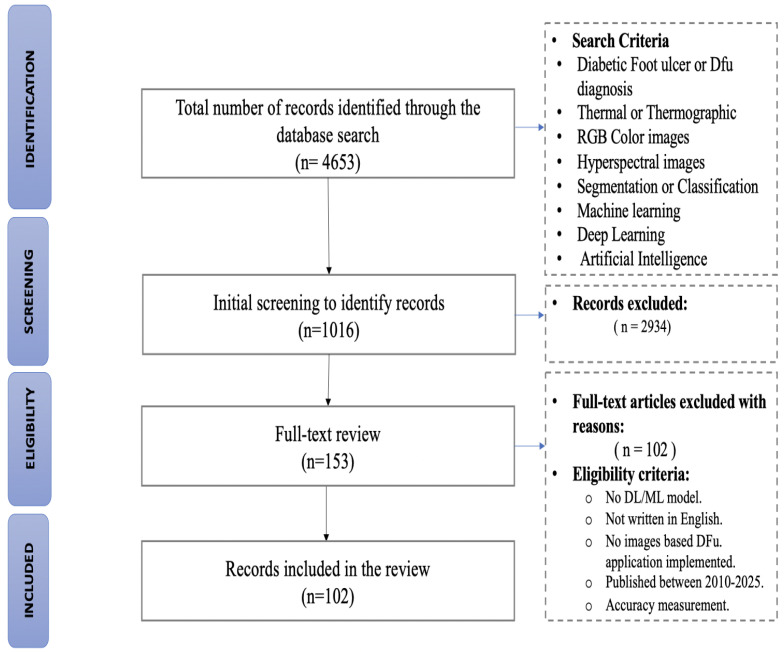
PRISMA flowchart of primary study selection. We excluded studies that have no ML or DL models, are not written in English, have no image-based model, or do not use accuracy measurements in the validation. However, we included studies that were published between 2010 and 2025. Records were excluded if the article is a preprint, has no AI application, search terms are not targeted in the article, or there are no accuracy measurements.

Full-length publications were retrieved in the context of the review from relevant journals. Two authors held a focus group to ensure that eligibility and inclusion criteria were fulfilled. A database of the identified literature, with titles, authors, publication dates, places of publication, and full abstracts was imported into Microsoft Excel. Duplicates were removed in software, and all other abstracts were screened by eligibility criteria. To begin with, each of the fourteen major bibliographic databases were searched. They were Web of Science, Scopus, PubMed, IEEE Xplore, and Science Direct. Other searches (e.g., Google Scholar, arXiv) were conducted to find suitable studies and to guarantee inclusivity. For search restriction, articles used were peer-reviewed, published in the English language, from 2010 to 2025. Preprints from databases such as arXiv were screened for background use and excluded during the screening phase. The search yielded 4653 articles, of which 2934 duplicates were excluded, and 1719 records were identified in the title and abstracts review. A total of 1566 articles were dropped out of the review due to the lack of inclusion criteria, leaving 1016 as the full-text for a detailed review. After reviewing the full text, 914 studies were excluded due to lack of AI applications or missing performance measures. In addition, 102 were included in the qualitative synthesis. The methods of study selection are provided in more detail in Figure 1.

As we considered the new trends in DFU detection using ML/DL techniques in DFU detection, we searched the following databases: Web of Science, Scopus, and PubMed between 2010 and 2025 considering the following topics: Diabetes Mellitus, Diabetic foot ulcers (DFUs), DFU dataset, Machine Learning (ML), Deep Learning (DL), Convolutional Neural Network (CNN), and Thermogram. The search was split between combinations of keywords using the “AND” connector: CNN AND DFU, DL AND DFU, ML AND DFU.

In total, 153 full-text papers from databases (Web of Science, Scopus, PubMed) were retrieved to perform the analysis, where 102 articles were selected for this review. The selection criteria were: (1) article published within a recent period, (2) presence of analysis of new trends in the field of ML/DL-based DFU detection, and (3) visibility and effect of the papers (published in a top-tier journal or conference and citation count). The article focus of the review was on a period ranging from 2010 to 2025 (i.e., 2018–2025, accounting for 94% of references as this is the most recent papers). Regarding recent developments in ML/DL-based DFU detection, segmentation and classification, citation counts were found to be varying according to publication date. In general, citation count in older papers was usually higher than a recent one (2022–2025), although there are a few interesting examples (e.g., refs. [27,28,31]). Hybrid neural networks (NNs) and transfer learning are emerging as alternative approaches in recent research, keeping pace with the latest developments in the space. Since new approaches were highlighted, no formal citation threshold was imposed in the selection sequence. Papers that followed the trends and where the number of cited papers seemed reasonable were the focus. Subsequently, we chose papers from the literature with the potential of being impactful and useful. More information about citation patterns and citation selections is given in Section 3. About 52% of the total references met this criterion. Several preliminary papers were reviewed by the focus group of the two authors above in order to ensure that relevant literature was not missed. For the systematic review and meta-analysis, we used a PRISMA (Preferred Reporting Items for Systematic Reviews and Meta-Analyses [32]) flow diagram.

### 2.4. Data Source

A comprehensive and reproducible search of electronic databases Science Direct, PubMed (MEDLINE), arXiv.org, MDPI, Nature, Google Scholar, Scopus, and Wiley Online Library was conducted to systematically identify relevant literature for this study (January 2010–October 2025). The common search terms, Boolean operators, and filters were used in all databases. Further detailed strategies based on keywords and specific queries per respective databases are also included in Supplementary File S2 for clearer visibility. The PRISMA flowchart (Figure 1) illustrates the study selection steps, and the removal of duplicates was carried out using reference management software to ensure accuracy.

We focused on terms related to diabetic foot ulcers and their diagnosis using machine learning and deep learning techniques. Keywords such as ‘classification,’ ‘detection,’ and ‘segmentation’ were included to ensure the search targeted studies specifically on image-based diagnosis, excluding unrelated AI applications in diabetes care. To enhance comprehensiveness, the search was expanded to include terms like ‘thermal imaging’ and ‘computer-aided diagnosis.’

**Figure 2:**
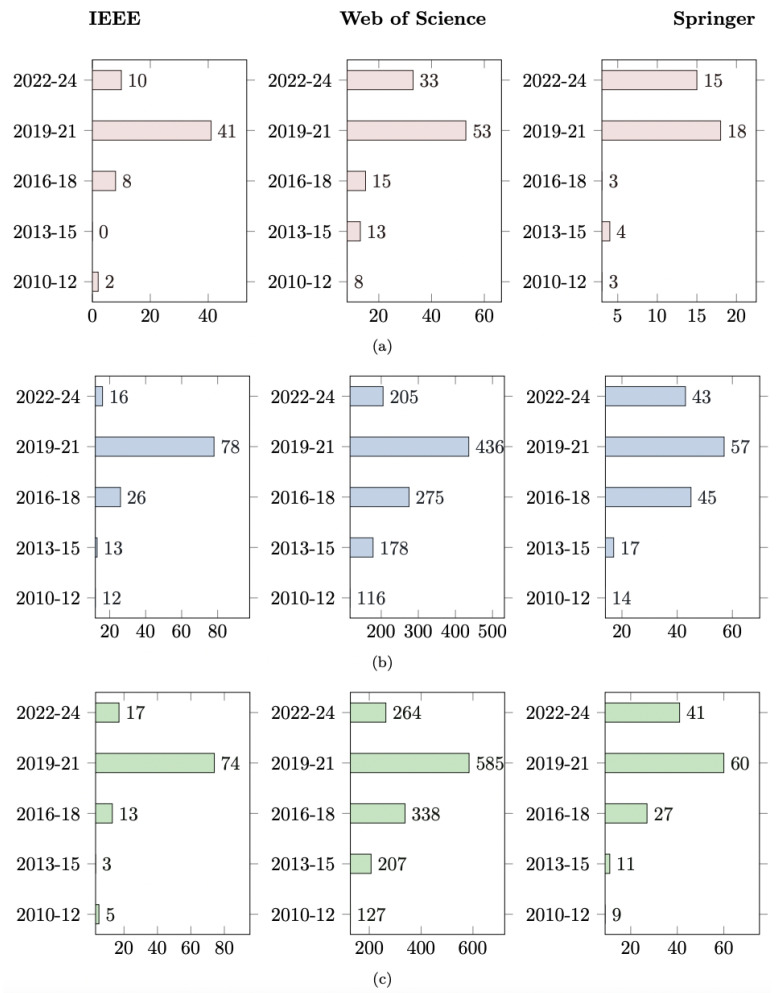
Term frequency trends in Web of Science, Scopus, and PubMed databases (2015–2022) using the AND connector: (**a**) CNN AND DFU, (**b**) ML AND DFU, and (**c**) DL AND DFU. Percentages are calculated based on the total number of retrieved records from each database for the specified time range.

A consistent set of predefined keywords and Boolean operators was applied across databases, using terms such as ‘Artificial Intelligence in DFU,’ ‘deep learning,’ ‘machine learning,’ ‘ANNs,’ ‘CNNs,’ ‘DFU detection,’ ‘DFU segmentation,’ and ’DFU classification.’ Additional filtering criteria, including publication years (2010–2025) and peer-reviewed articles, were applied to ensure relevance. Figure 2 illustrates the term frequency trends for DFU-related queries in Web of Science, Scopus, and PubMed databases between 2010 and 2025. Percentages are calculated based on the total number of retrieved records from each database during this period. Detailed database-specific queries and Boolean logic are provided in Supplementary File S2 for transparency. The search strategy used in this study is summarized in Table 1.

### 2.5. Assessment of Methodology Quality

QUADAS-2 was used to assess the methodological quality of the studies [33]. As a tool for evaluating the risk of bias and applicability of diagnostic accuracy studies, QUADAS-2 consists of four key domains: Patient selection, Index test, Reference standard, and Flow and timing.

In all four domains, bias risks were assessed, while concerns regarding applicability were raised in the first three. Concerns and risks were rated as high, low, or unclear. An unclear risk was determined when insufficient data were presented in the study. N/A was used when the QUADAS domain did not apply due to the study methodology. Any category with high levels of bias may indicate problems with the methodology of the paper, and high levels of bias across multiple categories may affect the validity of the reported results. A high risk of bias in terms of applicability in the tested domains may indicate that the included data from the evaluated paper do not accurately reflect the review question. The GRADE (Recommendation Evaluation, Development and Evaluation) approach is a systematic framework for evaluating the quality of evidence and the strength of recommendations in healthcare [34,35,36].

### 2.6. Diagnostic Accuracy Measures

In order to have a reasonable comparison, it is important to compare the analyzed papers based on their common statistical performance metrics. In detection, segmentation, and classification, a number of evaluation metrics are used, such as but not limited to Accuracy, Precision, Sensitivity, Specificity, F1-score, and Jaccard index. Table 2 shows the details of these metrics’ mathematical formulas.

### 2.7. Data Synthesis and Analysis

We categorized the extracted data according to the type of ML/DL functionality they were designed for (detection, segmentation, or classification). Furthermore, the data were classified according to the methodology used in each section, which was determined by the functionality of each section.

This enabled direct comparison of data between studies. Regardless of the measure used by the included papers, all outcome measurements were extracted and analyzed in a standard format, including all definitions of accuracy. However, due to variations in study designs, datasets, and evaluation methods, statistical pooling was not conducted. Instead, descriptive analysis of reported metrics (e.g., accuracy, AUC, sensitivity) was performed, and results were grouped by ML/DL functionality (detection, segmentation, classification, hybrid models) to enable meaningful comparisons. For comparison and understanding of ML/DL efficacy across similar tasks, each ML/DL model contains its respective outcome measure value.

Due to the inclusion of a range of terms related to accuracy in the search criteria, no papers were excluded based on any accuracy measurement. This is because they were not explicitly mentioned in the search parameters. The absence of accuracy measures in this review indicates the absence of relevant articles describing these statistical measures as outcomes. We illustrate the papers that met the criteria as sets of tables, distributed based on the functionality of the models. In addition, pie charts are used to visualize the proportion of model functionality across types of data and across available datasets. Furthermore, a bar chart provides a quantified demonstration of different models. Moreover, see Supplementary File S2 for full details about articles and how we treat them according to our inclusion and exclusion criteria.

## 3. Results

There were 2215 papers identified in total. Titles were initially screened to identify records, leaving 300 titles to be evaluated before applying exclusion criteria. Based on the eligibility criteria, 173 studies were excluded and the total number of included studies was 127. Many examples of excluded papers, such as [37,38], were excluded because no image-based DFU diagnosis was applied, and [31,39] were excluded due to lack of accurate information. This process is outlined in the flowchart below based on the PRISMA-DTA methodology. The 102 papers included in this review reported multiple forms of machine/deep learning.

**Figure 3:**
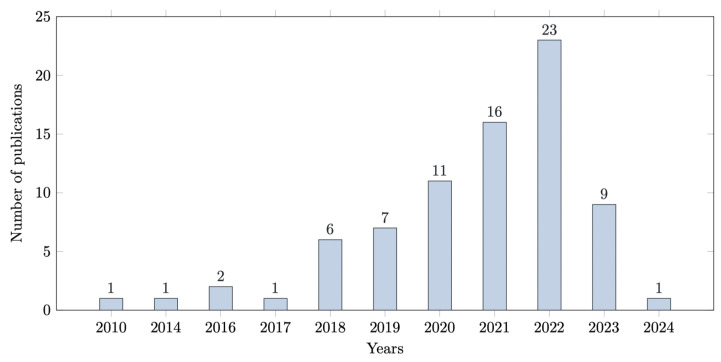
ML and DL in DFU research trends among included studies published between 2010 and 2025. Percentages are based on the total number of included studies (n = 102). The x-axis represents publication years, while the y-axis indicates the number of publications.

Hence, this paper includes 127 studies with a variety of characteristics and demographics. The full details of the included studies’ characteristics can be found in Section 5. Figure 3 shows the trends in ML and DL research for diabetic foot ulcers published between 2010 and 2025. The percentages are based on the total number of included studies (n = 102), and the x-axis represents the publication years.

**Figure 4:**
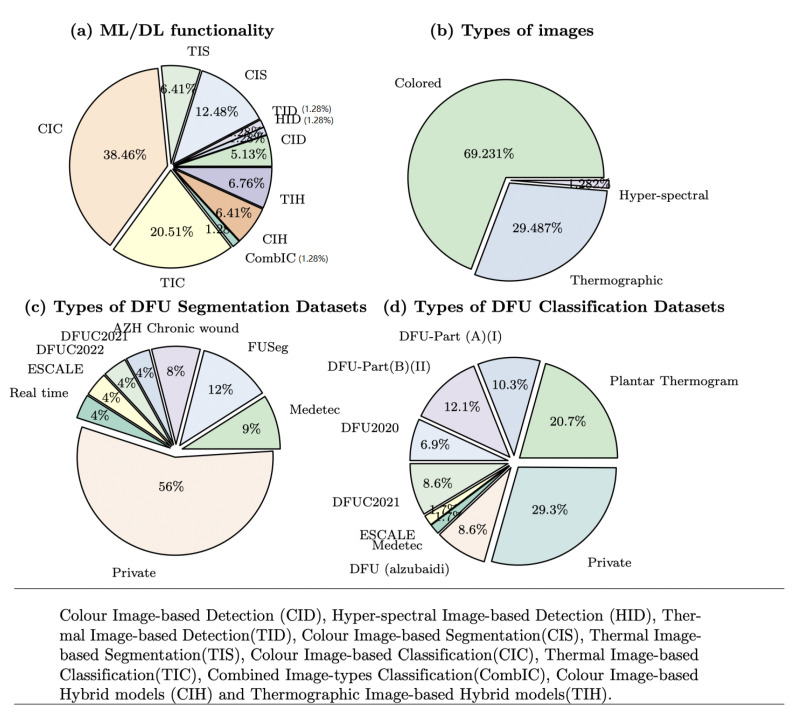
(**a**) Percentage of included studies (n = 102) by ML/DL functionality for DFU diagnosis. (**b**) Percentage of studies using different DFU image types (colored, thermal, hyperspectral). (**c**) Percentage of segmentation studies using public datasets. (**d**) Percentage of classification studies using public datasets. Percentages are calculated relative to the total number of included studies. Data spans the publication period of 2010–2025.

Figure 4a shows the proportional distribution of ML/DL functionality, where 38.46% focus on Color Image-based Classification (CIC). As shown in Figure 4b, most of the ML/DL models used colored images (69.23%) while only 29.49% used thermographic images and 1.29% used hyperspectral images. Datasets used across different functionalities varied as illustrated in Figure 4c,d. In total, 56% and 29.3% of the studies used different publicly available datasets for segmentation and classification models, respectively. Figure 4 shows that 56% of the studies in segmentation and 29.3% of the studies in classification used at least one publicly available dataset. This distinction between dataset availability and its usage highlights the need for better adoption of public datasets in research.

**Figure 5:**
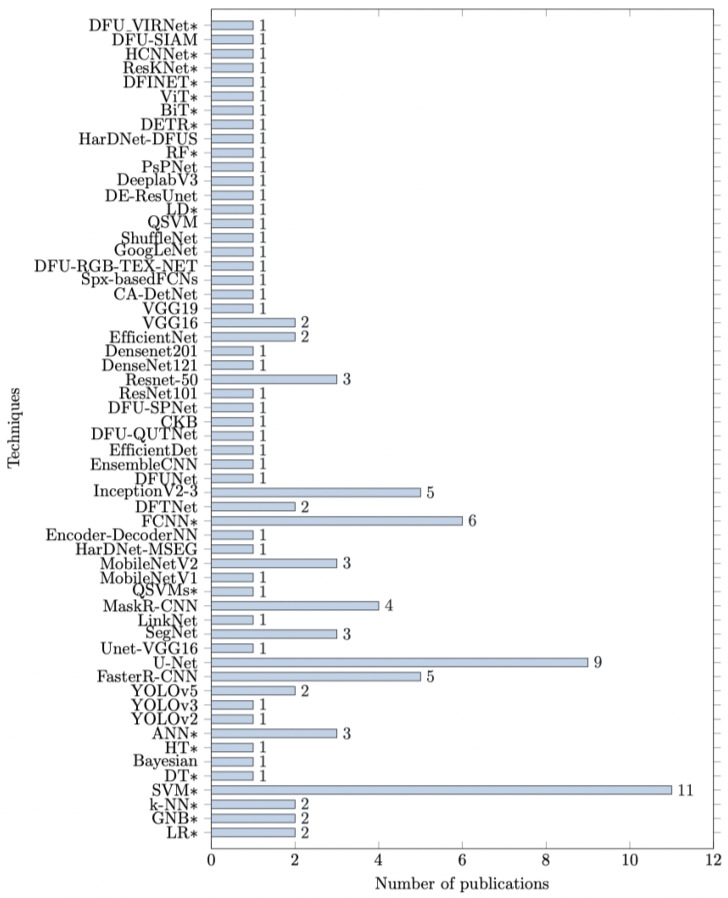
Graphical display of machine/deep learning references in included studies.

Figure 5 graphically illustrates the prevalence of using different architectures of ML/DL models in image-based DFU diagnosis.

There has been considerable interest in the topic of classifying DFUs from color images, as shown in Figure 4a. In some approaches, DFUs were classified by severity stage following the Wagner grading of ulceration using either a large or small dataset, while in others, the DFUs were classified by ischemia and infection recognition. Furthermore, other researchers performed the classification of normal and abnormal DFU skin.

The most notable growth areas in the detection, segmentation, and classification of DFUs from color or thermal images, as shown in Figure 4a,b can be summarized in the following points:

- In the detection of DFU domains based on color and thermal images:
  1. You Only Look Once (YOLO) and its extensions are the more notable algorithms in the detection domain [40,41].
  2. Most of the current work employing thermal images uses classical classifiers such as Support Vector Machine (SVM), k-nearest neighbour algorithm (k-NN), etc. [42].
- In the segmentation of DFU domains based on color and thermal images, approaches included:
  1. Using probability-based segmentation [43].
  2. Leveraging computer vision techniques as pre- or post-processing to support NNs in segmentation results (colored images [44,45,46] and thermal [47]).
  3. Utilizing transfer learning techniques to solve the problem of DFU segmentation [48,49].
  4. Investigating the feasibility of using DL techniques to segment wounds under conditions of small datasets [50] although DL-based methods for automating the segmentation of wounds are currently known to require large datasets for training.
  5. Using U-Net and Mask Region-based Convolutional Neural Network (R-CNN) and their extension versions which are the most popular networks in segmenting DFUs (For U-Net: colored [50,51,52,53,54], thermal [55,56], Fully Convolutional Neural Network (FCNN) [50,56,57,58,59,60,61,62] and Faster R-CNN [63,64,65,66,67]).
  6. Using Encoder–Decoder NNs which show greater performance (i.e., SegNet, DE-ResUnet) than other NNs using thermal images [56,58].
- In the classification of the DFU domains based on color images, approaches included:
  1. Employing transfer learning to DFUs classification [59,68,69].
  2. Combining handcrafted features with deep features [70,71].
  3. Employing Class Knowledge Banks (CKBs) to improve the performance of DL classification [72].
  4. Combining a pre-trained CNN model with automatic classifiers, which showed promising results [73].
  5. Focusing on diagnosing more accurately and making less subjective real-time decisions [62,74].
  6. Improving external validity of the existing models by avoiding overfitting certain data [75,76]
  7. Focusing on overcoming severe class imbalances [77] using extension strategy and use of synthetic images appears to improve classification results for less frequent classes significantly.
  8. Setting up challenges to enrich the field with data, data analysis, and ground truth annotation [69].
- In the classification of the DFU domains based on thermal images:
  1. Focusing on the gap of finding a way to Peripheral Arterial Disease (PAD), a circulatory disorder characterized by reduced blood flow to the limbs, which significantly increases the risk of diabetic foot complications [78].
  2. Improving the effectiveness of classification methods for detecting abnormal changes in plantar temperature by combined transfer NNs [79,80,81] to achieve higher accuracy.
  3. Determining the severity of diabetic foot complications by combining classical ML approaches such as Random Forest (RF) combined with CNNs [82,83].
  4. Combining NNs by fusions [84], which showed higher accuracy.
  5. Adopting Transformers, which offers new opportunities for predicting ulcer risk such as Vision Transformer (ViT) [85] and Detection Transformer (DETR) [86].
  6. Using ML and image processing-based algorithms to locate hotspots in the feet [87].
- In the classification of the DFU domains based on different images, combining untrained and pre-trained transferred NNs to the field gives high yields, providing consistency across all performance metrics [24].
- In the hybrid frameworks (segmentation and classification) of the DFU, approaches included:
  1. Combining knowledge-based transfer learning modules, which establishes a broader research area with more experimental opportunities [88,89] and was found to be promising.
  2. Utilizing computer vision techniques, such as color and texture analysis [90,91,92] or uncalibrated visual fitting techniques [93], which can significantly improve results.

### 3.1. Performance Metrics

A summary of model performance measures across each target study is shown in Table 3, Table 4, Table 5 and Table 6. The mean detection accuracy of the best performing algorithm per study was 0.97 ± 1.0. Segmentation models had a mean accuracy of 0.94 ± 0.1, classification models 0.93 ± 0.006, and 0.88 ± 0.06 for hybrid studies. The mean AUC was also calculated as 0.97 ± 1.0 for detection, 0.99 ± 0.001 for segmentation, 0.94 ± 0.001 for classification, and 0.89 ± 0.09 for hybrid studies. The performance metrics varied significantly across studies due to differences in datasets, evaluation methods, and model architectures. Mean and standard deviations for key metrics, such as accuracy and AUC, are presented in Table 3, Table 4, Table 5 and Table 6 for each functionality.

As a result of our evaluation of the best performing algorithms, we found that their detection sensitivity was an average of 0.95 ± 1.0. Segmentation models had a mean sensitivity of 0.91 ± 0.09 and classification models 0.92 ± 0.07; hyper methods displayed the lowest mean sensitivity with 0.89 ± 0.09.

In terms of specificity, segmentation had the highest mean specificity, with 0.95 ± 0.03 (range: 0.90–0.98), compared with classification, which had a mean specificity of 0.94 ± 0.04 (range: 0.89–0.97), and the hybrid method, which had a mean specificity of 0.93 ± 0.04 (range: 0.89–0.96). The detection approach had the lowest mean specificity, with 0.85 ± 0.02 (range: 0.82–0.90). These parameters were determined by taking the metrics of the best performing algorithms in each study and calculating the mean values and standard deviations.

The detection models presented good performance across all studies. The mean accuracy of detection was recalculated as 0.97 ± 0.03 with a range of 0.90–1.0. The mean AUC was 0.97 ± 0.02 (range: 0.92–1.00), sensitivity was 0.95 ± 0.02 (range: 0.91–0.98), and specificity was 0.85 ± 0.02 (range: 0.82–0.90). A study showed the average accuracy value for detection models was 0.97 ± 0.03 in that same metric. Standard deviation and range were adapted to account for variability within the ranges they were within 0.90–1.00. These results reflect the best algorithm per study, which may lean toward optimism over other algorithms. Performance values reported are distributed across different architectures and datasets, which indicates high variability. The recalculated metric indicates that detection models typically display high accuracy rates but are influenced by dataset-specific features and model architecture, leading to varying performance. Separate datasets and evaluation measures adopted by the studies highlight the need for standardized benchmarks in order to ensure richer comparisons may be made.

### 3.2. Quality Assessment

**Figure 6:**
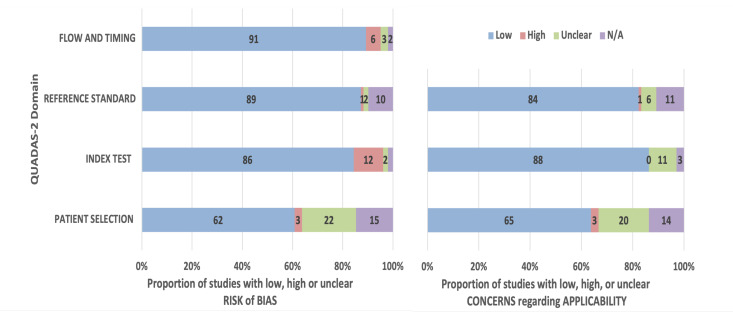
QUADAS-2 quality assessment graphs depict individual bias risk and concerns regarding applicability presented as percentages across the 66 included studies.

A variety of specific areas of the DFU have been assessed for the diagnostic accuracy of AI used throughout the studies. In order to assess the risk regarding bias, QUADAS-2 [33], a commonly used tool in the literature, was used. Overall, most studies were found to be low-risk with regard to bias and applicability. The current systematic review reported a low risk of bias in the index test used and in flow and timing (approximately 89% and 91%, respectively). However, there was a significant percentage of studies where the patient random selection domain was unclear in its risk of bias (22%) and applicability concerns (20%) or not applicable (15% for bias and 14% for applicability concerns). This is primarily due to a lack of published details regarding sample selection and clinical information about the patients. There was a high level of risk in flow and timing, around 6% of included studies, as a result of the small dataset used [42,91]. There was also a high risk associated with the selection of patients since it is unclear what criteria were used to select a random sample [40,56]. A comparable result was found for the applicability arm of the QUADAS-2, as shown in Figure 6.

Regarding the GRADE, an assessment of the studies reveals that most have few limitations; however, the presence of some studies with significant limitations (e.g., Study 1 and Study 68) pulls down the overall quality assessment of the evidence. In terms of consistency, most of the studies report similar findings, which supports the premise that there is quite a bit of reliable evidence here. Almost all of the evidence is directly applicable to the target population, but some of the evidence is indirect (e.g., Studies 1, 3, and 7). Several studies provided estimates of the effect that were precise. Several studies provided estimates of the effect that were imprecise and did not affect our overall confidence in the findings. The presence of several studies with both moderate and high certified study levels led to an overall rating of moderate certainty for this body of evidence. The details of the GRADE of each study can be found in Supplementary S2. Quality assessments.

### 3.3. Impact of Bias and Data Quality on DFU Model Performance

One stratified analysis shows that studies in which there is a high risk for bias or ambiguous reporting report inflated performance data. For example, detection models evaluated on private datasets with limited numbers of samples (e.g., less than 100 images) frequently demonstrated higher accuracy (>95%) relative to models tested on much larger and public data (e.g., DFUC2021), compared with 85–90% accuracy. This gap indicates that performance can possibly be overstated in studies with poorly defined patient selection and dataset characteristics. Such studies (for instance, ones in which the risk of bias is low, particularly when there are publicly accessible datasets such as DFUC2021 and Medetec; with standardized evaluation criteria) were able to achieve more uniform and reproducible performance. For instance, U-Net with clear validation protocols among segmentation methodologies had a Dice score of 94% on DFUC2021 while studies with non-standardized datasets or vague validation criteria had a Dice score that exceeded 98% but lacked reproducibility.

The GRADE recommendations were used in the quality of evidence for technical end-point studies in accuracy, AUC, Dice coefficient, and Intersection over Union. Methods that were based on low quality data from inadequate databases, suboptimal quality patient samples, or variation from approved protocol were reduced with consideration of bias. For example, studies conducted on private datasets were not usually transparent about dataset diversity or representativeness, leading to greater likelihood of overfitting and inflated performance claims. We also examined variation from studies in reported performance metrics to assess for inconsistency. One study using U-Net applied a segmentation model on DFUC2021 dataset, which reported Dice scores of about 94% throughout the datasets, whereas more studies utilizing non-standard datasets and unclear analytical methods reported Dice scores up to 98%. Because some studies also provided inconsistent data, the quality of evidence at some studies was downgraded. Table 7 summarizing GRADE judgment results for detection, segmentation, and classification tasks gives a summary of these and additional findings.

The significance of technical endpoints to clinical outcomes were also considered to account for indirectness. Although metrics like Dice coefficient and AUC are essential to evaluate model performance, they do not forecast clinical outcomes, such as ulcer healing or amputation avoidance. Therefore, studies excluding clinical endpoints received poorer scores in indirectness. Imprecision was also a variable that influenced evidence quality when studies with smaller sample sizes and large confidence intervals for key metrics were considered unvalidated. Studies that trained models on fewer than 100 images, for example, often reported very noisy performance measures and their findings were therefore not very trustworthy. In terms of these assessments, the quality of evidence for the detection model in total was moderate according to the same criteria used in previous studies. This was found to be steady for all studies performed using the available public datasets. The validation evaluation of classification studies was weak, with a low-to-moderate ranking because of the fact that datasets used were small, and there was a dearth of uniformity of dataset and high variability.

## 4. AI in Diabetic Foot Management

AI has generated a widespread revolution in medical imaging over the past ten years, including but not limited to X-ray, ultrasound, computed tomography, and magnetic resonance imaging. However, AI-based systems for high-quality wound care remain significantly underdeveloped in both clinical and computational domains [94,95]. Initiatives to implement AI for optimizing data processing and products for diabetes management appear to be ongoing and carry great potential for the near future [96]. The progression of this AI revolution could be broken down into a number of stages: through the introduction of Neural Networks (NNs), the development of various ML algorithms, and most recently the current era of DL.

Hazenberg et al. [97] performed a comprehensive review of the PubMed database to identify the nature, functionality, efficacy, cost, and present limitations of telehealth and telemedicine applications related to diabetic foot disease prevention and management. They found that such applications are still in the early stages of development, and stronger scientific evidence is necessary to support efficacy and feasibility of such applications. Next, a more technically and economically efficient system would need to be built before such systems are widely deployed in patients’ homes. In terms of applicability, Tulloch et al. [11] confirm through using PRISMA-DTA at PubMed, Google Scholar, Web of Science, and Scopus for ML algorithms in DFU studies that the current research is limited and that there is a need for the development of more applicable ML algorithms. In terms of characterization, in [94], provide a wide view of the literature is provided on the effect of developing novel Artificial Intelligence (AI) systems that can help clinicians diagnose, assess the effectiveness of therapy, and predict healing outcomes.

Moreover, the COVID-19 pandemic posed significant challenges to diabetic foot clinics since most patients were unable to physically attend clinics. So, the pandemic underscored the urgent need for AI-based wound care to monitor DFUs remotely. As a result, virtual clinics gained popularity [98]. This review focuses on ML/DL literature for three primary purposes: detection, segmentation, and classification.

## 5. ML/DL Models Used in Diabetic Foot Detection, Segmentation, and Classification

One of the earliest AI experimental of applying AI to DFU detection and segmentation was by Wang et al. (2016) [27,99]. In this study, images taken in a special capture box were analyzed with wound image assessment algorithms to calculate the overall wound area, color-segmented wound areas, and a healing score. The quantitative assessment of wound healing status can be obtained from these measurements. A Support Vector Machine (SVM) was used to locate wound borders on foot ulcer images. However, this method also comes with significant drawbacks, including physical contact between wounds and the capture box and the danger of contamination. Later, the work of Goyal et al. [57,63,68,100] trained different models capable of classification, detection, and segmentation. Detailed information about these models can be found in Section S1 of the Supplementary File S1. Furthermore, Supplementary File S1, Section S2 provides detailed information about the DFU images and datasets used in the literature.

Table 8 summarizes the main characteristics and outcomes that were measured in the included detection-targeted studies. Table 9 shows the details of segmentation techniques described in the papers included in this study and their characteristics. Table 10 summarizes the main characteristics and outcomes that were measured in included classification-targeted studies. Table 11 summarizes the main characteristics and outcomes that were measured in the included hybrid (classification and segmentation).

## 6. Discussion

This section discusses the findings for each of the research questions in this review. The aim of this survey was to visualize the state of the art of ML/DL in DFU diagnosis, which can be described to include detection, segmentation, classification, or a hybrid system combining two or more of these functionalities.

Networks are heterogeneous in terms of their size, computation, and structural complexity. It is theoretically possible that deeper networks perform better than shallower networks, but in practice, deeper networks underperform shallower ones. This is due to an optimization problem rather than an overfitting problem. In general, the deeper a network, the more challenging it is to optimize it. This study shows that U-Net, FCNN, Faster R-CNN, and their related versions are the most popular networks for segmenting DFUs (for U-Net: colored [50,51,52,53,54], thermal [55,56] FCNN [50,56,57,58,59,60,61,62] and Faster R-CNN [63,64,65,66,67]).

Additionally, Encoder–Decoder NNs show outstanding segmentation performance (i.e., SegNet, DE-Resnet) among other NNs using thermal images [56,58] due to their architecture being based on utilizing low-resolution feature mapping. In fact, the proposed DE-ResUnet contains two encoders and one decoder, and each unit of the decoder consists of an up-sampling block followed by a convolution operation to produce dense feature maps which combine features and background knowledge that effectively identifies wound boundaries, improves accuracy, and enhances learning abilities which have been shown to provide superior segmentation performance.

Examples of the application of transfer learning to DFU classification (colored images [59,68,69], thermal [79,80,81]) demonstrte that transfer learning is a viable method for reusing the architecture and weights of a model trained with large amounts of input data and applying it to different scenarios and other datasets while using a lower amount of computational power. With respect to the work of Cassidy et al. [66], there is no study that has applied different architectures to the same dataset. Additional exploration is necessary to determine the advantages of transfer learning in DFU diagnosis. Therefore, future work should include an analysis of most transfer learning architectures as well as the self-tuning paradigm applicable to this field.

Regarding detection, YOLO family [151] architecture is one of the most popular models for DFU colored-imaging detection. The main reason for its popularity is that it utilizes a highly reliable and efficient neural network architecture. A variety of NN architectures were employed in the studies included in this literature review (see Figure 5).

Furthermore, we believe that the models with hybrid data use will make a significant contribution to the field. Recent work focused on creating integrated frameworks that blend two different modalities in one pipeline (thermal and color images [56]) which has helped to achieve remarkable segmentation accuracy.

At present, there is no publicly available dataset that contains multimodality imaging of diabetic foot patients. Obtaining such datasets requires a considerable amount of time and effort, as these datasets use a variety of imaging methods. For example, these include thermal infrared imaging (useful for detecting ulcers early), clinical wound imaging (for assessing ulcer progression), fluorescence (to check for the presence of clinically significant bacteria), and MRI (to detect Charcot’s foot and infection). According to the accuracy measurement display in Table 8, Table 9, Table 10 and Table 11, thermal infrared imaging has proven to be an effective tool in the clinical management of DFU patients.

Since the metrics reported were heterogeneous, it was difficult to compare model performance. The generally agreed validation measures among most of the included studies are the validation parameters from the confusion matrix: Accuracy (41 studies), Precision (27 studies), Specificity (21 studies), Sensitivity (Recall) (36 studies), DSC (32 studies), and AUC (9 studies) as shown in Table 8, Table 9, Table 10 and Table 11. The heterogeneity in performance metrics, due to differences in datasets, imaging modalities, and reporting practices, limited direct statistical comparisons. This highlights the need for standardized evaluation metrics and consistent reporting frameworks to improve comparability across studies. Adding the AUC (ROC) to these measurements provides the most effective method of reducing heterogeneity in the validation parameters. Ideally, reporting the same measurements throughout the AI revolution of DFU diagnosis will facilitate tracking the development of AI-guided diagnosis systems. To this end, a generalized standard assessment for DFU diagnosis applications can be developed in the future with further investigation.

In many research studies, colored images as shown in Figure 4b were used by 67% of selected studies but thermal infrared imaging has been proven to be a useful technique in the clinical management of DFUs [42,47,55,56,58,60,81,85,126,135,152]. Another imaging modality, known as hyperspectral fluorescence imaging, can detect clinically significant bacteria in diabetic foot ulcers, but little research has been conducted on this type of imaging [101]. Potentially, research can provide valuable information on DFU outcomes of the severity of a DFU or through the healing process. Moreover, MRI and CT are other options that can be used to investigate the DFU diagnosis process as proved by the feasibility of their use in other diagnoses in medical decision-making [153,154,155]. Future work in DFUs will include the use of such data in our follow-up work to ensure more accurate monitoring and timely treatment. While 45% of distinct segmentation datasets and 67.3% of distinct classification datasets are publicly accessible, only 45% of segmentation studies and 32% of classification studies used at least one publicly available dataset. This discrepancy underlines the need to encourage researchers to adopt publicly available datasets to improve the reproducibility and comparability of findings.

In terms of multimodality, there has been an effort to create multimodel DFU diagnosis systems such as in [24], but there are no publicly available datasets that combine multi-modality imaging of diabetic feet. The collection of such datasets combining IRT, clinical DFU images, fluorescence, and MRI requires a great deal of effort. As with other medical imaging datasets, DFU datasets often exhibit image duplication, and feature over-representation and excessive reliance on a small number of subjects [21].

Using machine learning for Diabetic Foot Ulcers (DFUs) is not excluded from the challenges and proposed solutions. Data scarcity and data quality can be improved by handling them in a variety of ways such as using synthetic augmentation methods like GAN generative adversarial networks or collaborating with medical institutions. Three options are available to alleviate class imbalance among healthy and ulcerated images: oversampling, undersampling, and weighted loss functions. The wide heterogeneity of ulcers demands comprehensive feature selection and an ensemble model for accurate prediction. Interpretability is key, so the utilization of interpretable models or methods such as SHAP and LIME can assist in elucidating decisions to clinicians. Making it more user-friendly and interoperable with health records is critical to integration into clinical workflows. In order to improve generalization onto other populations, it is important that the model be trained on diverse datasets, and domain adaptation techniques are required. Transfer learning using pre-trained models can improve the performance with less data, and federated learning makes it possible to collaborate without sharing sensitive information. Alzubaidi et al. [156] adapted a pre-trained skin cancer model to classify foot skin images into two categories: normal or abnormal (diabetic foot ulcer) to overcome the limitation of data availability.

### 6.1. Addressing Research Questions: Models, Metrics, and Dataset Characteristics

This study evaluated the effectiveness of ML/DL models, optimal validation metrics, and the impact of dataset characteristics on DFU diagnosis. For RQ1, U-Net obtained the highest performance for segmentation tasks, with the Dice coefficient of 94% and IoU of 89% on the DFUC2021 dataset, compared with Mask R-CNN (Dice: 91%). EfficientNet achieved 99% accuracy in the classification for DFUC2020; YOLOv5 achieved the highest mAP of 92% in DFUC2020. These findings suggest the relevance of choosing task-dependent models validated with standardized datasets.

Regarding validation metrics (RQ2), task-specific measurements are critical. When it comes to detecting, mAP and localization accuracy are appropriate for conducting the performance validation of the model, as YOLOv5 achieved 92% mAP. Dice coefficient and IoU are useful features for segmentation tasks, and U-Net attained a Dice score of 94% on DFUC2021. Classification models use AUC, accuracy, and precision metrics, with EfficientNet achieving an AUC of 99% on DFUC2020. Measurement metrics such as decision curves can further help enhance clinical relevance.

For dataset characteristics (RQ3), model generalization would improve with larger and more varied datasets. With over 4000 images, DFUC2021 generated better performance when compared with smaller datasets such as Medetec (<1000 images). High-quality annotations (pixel-level masks, DFUC2021) were one of the many enablers for the powerful segmentation performance of U-Net. Yet, class imbalance is an issue, especially for classification tasks. Methods like oversampling, weighted loss functions, and GAN synthetic data generation are known for mitigating this problem. U-Net, EfficientNet, and YOLOv5 are the most efficient models for segmentation, classification, and detection (respectively) on multiple, higher quality datasets if used based on task-specific metrics. Managing dataset constraints and deploying metrics in common are vital to improving ML/DL-based DFU diagnosis.

Transformers including ViT and DETR provide opportunities in developing predicting ulcer risk due to their capability to exploit global relationships in image data. It is in line with RQ1 as these architectures enhance diagnostic accuracy and generalizability. Moreover, self-supervised learning approaches with little labeled data can respond directly to deficiencies in data size and annotation costs mentioned in the Results. These techniques are specifically appropriate for Research Question 3 (RQ3) since they decrease reliance on large labeled datasets and lead to strong model performance.

### 6.2. Limitation of Included Research

Although filters are not recommended in systematic review methodology, the limits applied by the authors of this study are unlikely to have influenced the articles retrieved. The limits were considered satisfactory in order to limit the number of irrelevant articles without impacting the retrieval of relevant articles. The review question and eligibility criteria were limited to human studies. Considering the scope of this review, we included only papers published between 2010 and 2025, as well as the number of publications, which is unlikely to have had an impact on the results. In addition, the key limitation of this review is the exclusion of studies in usability, implementation, or clinical decision-making metrics. These are important to real-world adoption and use cases, and the focus of this review was intended to remain narrowly directed at diagnostic performance in image-based activities. This review acknowledges this limitation, and the findings might be extended by investigating usability, implementation related studies to better understand the challenges and opportunities of machine learning/deep learning models for clinical use. This clarification was developed based on reviewer feedback regarding the possible consequences of inclusion and exclusion criteria.

### 6.3. Fairness, Generalization, and External Validation

Fairness in diabetic foot imaging has attracted attention in particular, with works like Reis et al. [147], which showed that the ML/DL models had differential performance on different skin tones. These models learn on poorly represented datasets and thus their performance is poor on individuals with darker skin tones. All of these data collection biases must be mitigated by having diverse and inclusive datasets. Furthermore, the generalization to clinical centers and geographic regions of these models is still problematic because of differences in the imaging protocols and the patients’ demographics. Such a model robustness can be enhanced through multi-center datasets and federated learning approaches. External validation on geographically and demographically diverse cohorts is needed to ensure the reliability of these research studies, especially since most rely on a single-center or private dataset. Upcoming initiatives should emphasize diverse datasets, collaborative efforts, and fairness audits to optimize equity and generalizability of DFU diagnostics.

## 7. Conclusions

There has been rapid growth in ML applications for DFU, particularly with DL models that report high accuracy but lack publicly available datasets. To achieve significant results, denser CNNs are being developed to enhance the impact of DFU diagnosis in the future. To boost productivity for machine learning/deep learning technologies, it is essential to gather vast amounts of data. The diverse examples offered by big data help models learn to ‘see’ new data better. In the field of model training, more data means the models can work within better approximations—that is, they can figure out what ‘features’ in the data are important and can perform better under a set of validations. The idea here is to leverage this model training to make the automated diagnosis of DFU more efficient. Future research should include the following to enhance reproducibility, generalizability, and standardization: (1) creating and validating multimodality imaging of diabetic foot datasets, (2) training and testing on multimodality datasets alongside thermal image datasets, as they prove feasible in diagnosis, and reporting a full set of performance metrics, and (3) adhering to Wagner grading of ulceration to standardize DFU diagnosis. Therefore, we summarize our general recommendation from this review as follows:

- Development of Public Benchmarks: Establish centralized, publicly accessible repositories of multimodal DFU imaging datasets annotated with clinical metadata and severity grading (e.g., Wagner scale).
- Standardized Evaluation Metrics: Promote the consistent use of metrics such as area under the curve (AUC), Dice similarity coefficient (DSC), and Jaccard index to ensure comparability and facilitate meta-analyses.
- Community-driven Benchmarking Initiatives: Encourage reproducibility through organized challenges (e.g., DFU Grand Challenge) that provide standardized tasks and evaluation protocols.
- FAIR Data Principles: Ensure that datasets adhere to FAIR principles (Findable, Accessible, Interoperable, Reusable) to support long-term usability and collaboration.
- Reporting Standards: Recommend adoption of AI-specific reporting guidelines such as CONSORT-AI and PRISMA-DTA in DFU-related research publications.

This review has several implications for key stakeholders. For clinicians, it highlights the growing reliability of ML/DL tools in DFU diagnosis and underscores the need for clinical collaboration in validating AI models with real-world data. For data scientists, the review emphasizes the importance of developing models that are not only accurate but also interpretable, reproducible, and trained on diverse, multimodal datasets. For policymakers, the findings support the establishment of open data initiatives, regulatory standards for AI in wound care, and funding mechanisms to promote the development and deployment of equitable and evidence-based AI solutions in diabetic foot management. Future research should adopt standardized and consistent evaluation metrics, such as AUC and the Dice coefficient, to address inter-study heterogeneity and facilitate robust meta-analyses.

## Figures and Tables

**Table 1:** The research strategy followed in this study.

Database	Search Strategy	Search Data	# of Identified Records
IEEE Xplore	‘Diabetic Foot Ulcer OR DFU Thermal’ or ‘Thermographic’ ‘Segmentation or Classification’ AND ‘Machine learning’ OR ’Deep Learning’ OR ‘Artificial Intelligence’ ‘Foot Wound Tissue’ OR ‘Diabetic Foot Infections’ ‘Foot Ulceration’ OR ‘Chronic Wound Analysis’ ‘Foot diagnosis OR diabetic foot care’ ‘intelligence’ OR ‘Full Text OR Paper’ ‘Title’ OR ‘Survey’ OR ‘Overview’	15 October 2025	195
Science Direct			708
PubMed (MIDLINE)			1200
arXiv.org			60
MDPI			174
IEEE			160
PloS			60
Nature			172
Scopus			931
Springer			682
Elsevier			334
Taylor & Francis			69
Frontiers			46
Wiley Online Library			78

**Table 2:** Summary of statistical performance indicators used in the analyzed papers.

Metrics	Formula	Definition
Accuracy	$\frac{TP+TN}{TP+TN+FP+FN}\;\left(1\right)$	The accuracy of a measurement can be demonstrated by how closely it resembles the actual value or a standard.
Precision	$\frac{TP}{TP+FP}$	Precision is an indicator of how closely two or more measurements are aligned with each other.
Recall (Sensitivity)	$\frac{TP}{TP+FN}$	It quantifies how many correct positive predictions were made out of all possible positive cases.
F1 score (Dice Similarity Coefficient (DSC))	$\frac{2\cdot TP}{2\cdot TP+FP+FN}$	It is biased towards the lowest precision and recall values in each category. The F1 score increases if both precision and recall improve.
Specificity	$\frac{TN}{TN+FP}$	An indicator of the likelihood of a negative test being correctly identified (true negative rate).
Jaccard index (Intersection over Union)	$\frac{TP}{TP+FN+FP}$	This index measures the degree of similarity between two sets of members to determine which members are similar and which are different.
Five-fold cross-validation	$cv\left(k\right)=\frac{1}{k}\sum_{i=1}^{k}MSE_{i}$	It averages the Mean Squared Errors (MSEs) across *k* folds to detect overfitting and assess generalization performance.
Intersection over Union (IoU)	$IoU=\frac{\left|A\cap B\right|}{\left|A\cup B\right|}$	IoU measures the overlap between the predicted segmentation (*A*) and the ground truth segmentation (*B*), divided by their union. This metric evaluates segmentation accuracy, with higher IoU values indicating better performance.
Root Mean Square Error (RMSE)	$\sqrt{\frac{\sum_{i=1}^{N}{\left(x_{i}-{\hat{x}}_{i}\right)}^{2}}{N}}\;\left(2\right)$	It is the square root of the Mean Squared Error (MSE) of an estimator of a population parameter.
Area Under Curve (AUC)	–	An AUC measure is used to determine the entire two-dimensional area underneath the entire ROC curve.
Receiver Operating Characteristic curve (ROC)	–	It shows the performance of a classifier at all classification thresholds.
Error rate	$\left[\frac{\mathrm{Approximate}\;\mathrm{Value}-\mathrm{Exact}\;\mathrm{Value}}{\mathrm{Exact}\;\mathrm{Value}}\right]\times 100$	An approximate or measured value is expressed as a percentage of an exact or known value.
Mean Average Precision (mAP)	$\frac{1}{n}\sum_{k=1}^{n}AP_{k}\;\left(3\right)$	The metric is commonly used for evaluating the detection and classification of objects (i.e., localization, classification).
Kappa index	$\frac{P_{0}-P_{e}}{1-P_{e}}\;\left(4\right)$	It measures the level of inter-rater reliability between categorical variables.
Matthews Correlation Coefficient (MCC)	$\frac{TP\times TN-FP\times FN}{\sqrt{\left(TP+FP)(TP+FN)(TN+FP)(TN+FN\right)}}$	It measures the difference between the expected and actual values.
False Positive Rate (FPR)	$\frac{FP}{FP+TN}$	The proportion of incorrect positive predictions out of all actual negative cases.
Overlap score	$\mathrm{overlap}\left(X,Y\right)=\frac{\left|X\cap Y\right|}{\min(\left|X\right|,\left|Y\right|)}$	An overlap between two finite sets is measured by this similarity measure.
Success rate	$C=\frac{x}{y}*100\;\left(5\right)$	It is referred to as the success fraction when the success rate is determined based on the number of attempts.
System Usability Scale (SUS)	–	It is a commonly used method for measuring perceived usability of products and services, consisting of a 10-item questionnaire on a Likert scale, with participants answering each of the 10 items on a five-level scale.

(1) TP is true positive, TN is true negative, FP is false positive, and FN is false negative cases; (2) *N* is the number of data points, *x_i_* are real-time series observations, and ${\hat{x}}_{i}$ refers to time series estimates; (3) *AP* is the Average Precision, *k* represents an individual class, and *n* represents the total number of classes; (4) *P*_0_ indicates the relative agreement among raters, and *P_e_* indicates the hypothetical probability of chance agreement; (5) *C* is the chance of success or failure, *x* is the number of successes or failures, and *y* is the total number of attempts.

**Table 3:** A summary of the mean (±standard deviation) overall performance metrics for detection studies.

Accuracy (n = 1)	AUC (n = 1)	Sensitivity (n = 1)	Specificity (n=1)
0.97 ± 0.03	0.97 ± 0.02	0.95 ± 0.02	0.85 ± 0.02
(0.90–1.00)	(0.92–1.00)	(0.91–0.98)	(0.82–0.90)

n indicates the number of studies reporting metrics.

**Table 4:** A summary of the mean (±standard deviation) overall performance metrics for segmentation studies.

Accuracy (n = 8)	AUC (n = 2)	Sensitivity (n = 5)	Specificity (n = 4)	IoU (n = 7)
0.94 ± 0.05	0.99 ± 0.01	0.91 ± 0.04	0.95 ± 0.03	0.87 ± 0.07
(0.85–0.99)	(0.96–1.00)	(0.88–0.96)	(0.90–0.98)	(0.80–0.95)

n indicates the number of studies reporting metrics.

**Table 5:** A summary of the mean (±standard deviation) overall performance metrics for classification studies.

Accuracy (n = 26)	AUC (n = 6)	Sensitivity (n = 16)	Specificity (n = 15)
0.93 ± 0.04	0.94 ± 0.03	0.92 ± 0.05	0.94 ± 0.04
(0.88–0.98)	(0.90–0.98)	(0.87–0.96)	(0.89–0.97)

n indicates the number of studies reporting metrics.

**Table 6:** A summary of the mean (±standard deviation) overall performance metrics for hybrid (classification and segmentation) studies.

Accuracy (n = 7)	Sensitivity (n = 3)	Specificity (n = 3)	DSC (n = 1)	SUS (n = 1)
0.88 ± 0.05	0.89 ± 0.06	0.93 ± 0.04	0.94 ± 0.02	0.88 ± 0.02
(0.80–0.93)	(0.83–0.95)	(0.89–0.96)	(0.92–0.96)	(0.86–0.90)

n indicates the number of studies reporting metrics.

**Table 7:** GRADE Judgments for included studies by task.

Task	Risk of Bias	Inconsistency	Indirectness	Overall Evidence Quality
Detection	Moderate	Moderate	High	Moderate
Segmentation	Low	Low	Moderate	High
Classification	High	High	High	Low to Moderate

**Table 8:** Main characteristics of included detection-targeted DFU studies including author, year, journal ranking, dataset employed, resolution of images used, used ML model, validation metrics, and their results.

Author [Refs.]	Year	Journal Rank (SJR)/Conference Rank (Qualis)	ML/DL Model	Dataset	Validation Parameter	Value
Dremin et al. [101]	2021	Q1	ANN	Private Hyperspectral images dataset	Sensitivity, Specificity, AUC	0.95, 0.85, 0.97
Nag et al. [42]	2021	Not Yet Assigned	SVM, k-NN, and DT	PLANTAR THERMO-GRAM	Accuracy	97.778%
Cassidy et al. [64]	2022	Q1	Faster R-CNN	Real-time images	NAN	NAN
Thotad et al. [102]	2023	Q1	EfficientNet	DFUC2020	Accuracy, F1-score, Recall, Precision	98.97%, 98%, 98%, and 99%
Sarmun et al. [103]	2024	Q1	Combined Deep Learning Models	DFUC 2020	Localization Accuracy	86.4%
Sendilraj et al. [104]	2024	Q1	DFUCare Platform	DFUC 2020	Usability (F1-score, mAP, Ischemia, Infection)	F1: 0.80, mAP: 0.861, Ischemia: 94.81%, Infection: 79.76%
Biswas et al. [105]	2024	Q1	XAI-FusionNet	DFU Dataset (Kaggle)	Accuracy, Transparency	Accuracy: 99.05%, Precision: 100%, Recall: 98.18%, AUC: 99.09%
El-Kady et al. [106]	2024	Q2	ResNet + Generative Adversarial Network(GAN)	Clinical Dataset (Egypt)	Precision, F1-score	Precision: 0.85, F1-score: 0.84
Azeem et al. [107]	2024	Q2	SSD and YOLO Architectures	Clinical Dataset	Optimization Performance	Improved Detection (Exact values not mentioned)
Verma [108]	2024	Q2	Smart Image Processing Techniques	Thermal Dataset	Early Detection	ResNet50: 89.1%, EfficientNetB0: 99.4%
Busaranuvong et al. [109]	2024	Q1	ConDiff (Guided Conditional Diffusion Classifier)	Infection Prediction Dataset	Prediction Accuracy	Enhanced Accuracy (Exact values not mentioned)
Eldin et al. [110]	2025	Q1	Deep Neural Networks (ORB + DL)	Plantar Thermogram Dataset	Accuracy, F1-score, AUC	Accuracy: 98.51%, F1: 98.97%, AUC: 1.00
Rathore et al. [111]	2025	Q1	Feature Explainability-Based Deep Learning	DFU_XAI Dataset	Interpretability, Accuracy	Accuracy: 99.05%, AUC: 0.99, Precision: 100%
Debnath et al. [112]	2025	Q1	Sustainable AI with Deep Learning	DFUC 2020	Early Diagnosis, Resource Efficiency	DenseNet: 92.2%, MobileNet: 95.4%, FusionNet: 97.8%
Mahmud et al. [113]	2025	Q1	DFU_DIALNet (Grad-CAM + LIME)	DFU Dataset (Clinical)	Reliability, Trustworthiness	Grad-CAM Accuracy: 98.76%, LIME: Improved Explainability
Girmaw et al. [114]	2025	Q1	MobileNetV2	Ethiopian Hospital Dataset	Detection and Grading	Accuracy: 100%, AUC: 1.00
Pradhana et al. [115]	2025	Q2	CNN + SMOTE-IPF	Thermogram Images	Detection on Imbalanced Data	AHE Accuracy: 99.60%, Gamma Correction: 98.80%

**Table 9:** Characteristics of segmentation-targeted DFU studies including author, year, journal ranking, dataset employed, resolution of images used, used ML model, validation metrics, and their results.

Author [Refs.]	Year	Journal Rank (SJR)/Conference Rank (Qualis)	ML/DL Model	Dataset	Validation Parameter	Value
Wang et al. [99]	2016	Q1	SVM	Private dataset contain 100 foot ulcer color images	Sensitivity, Specificity	73.3%, 94.6%
Cui et al. [43]	2019	B1	CNN, SVM	The dataset contains 445 images 392 images for validation and 53 images for testing	Precision, Sensitivity, Specificity, Accuracy, Mean IoU, Dice and MCC	0.722%, 0.9%, 0.947%, 0.934%, 0.660%, 0.770% and 0.753%
Gamage et al. [116]	2019	Not Yet Assigned	Mask-RCNN (Backbone = ResNet-50, ResNet-101)	Private dataset has 2400 images	Average Precision, IoU	(ResNet-50 = 0.44, 0.51) (ResNet-101 = 0.51, 0.62)
Ohura et al. [51]	2019	Q2	U-Net and VGG16	Sacral Pressure Ulcers (PU) datasets	AUC, Specificity and Sensitivity	0.997%, 0.943% and 0.993%
Rania et al. [50]	2020	C	U-Net	ESCALE	Accuracy, IoU and DSC	94.96%,94.86% and 97.25%
Munoz et al. [48]	2020	Q2	Mask R-CNN	Private dataset	Accuracy, Sensitivity, Precision, Specificity and F Measure	98.01%, 96.97%, 97.94%, 95.97% and 97.01%
Bouallal et al. [55]	2020	B4	U-Net	Private dataset	IoU and DSC	Multimodal images: IoU = 98.37% and DSC = 99, Thermal data: IoU=97.43% and DSC = 98.68%
Mahbod et al. [53]	2021	A1	U-Net and LinkNet	Private dataset	DSC, Precision, Recall and IoU	84.42, 92.68, 91.80, 85.51
Galdran et al. [54]	2021	A1	Double Encoder-ResUnet (DE-ResUnet)	Private dataset	Precision, Recall and DSC	90.03%,86.91% and 84%
Chitra et al. [117]	2022	Q4	Random Forest algorithm (RF)	Private dataset	Accuracy	93.8%
Heras et al. [46]	2022	Q4	Logistic Regression(LR), morphological operators	Private dataset	Jaccard Index, accuracy, recall, precision and DSC	0.81, 0.94, 0.86, 0.91 and 0.88
Bougrine et al. [58]	2022	Q1	FCN, SegNet, U-Net	Private dataset	RMSEand DSC	5.12 pixels and 94%
Bouallal et al. [56]	2022	Q3	FCN, SegNet, U-Net	Private dataset	IoU	97%
Chang et al. [118]	2022	Q1	U-Net, DeeplabV3, PsPNet, FPN and Mask R-CNN)	Private dataset	Precision, Recall and Accuracy	(DeeplabV3 = 0.9915, 0.9915, 0.9957) in classification, (DeeplabV3 = 0.9888, 0.9887, 0.9925) in segmentation
Jain et al. [79]	2022	Ph.D. Thesis	ProNet	Private dataset	Accuracy	98.9%
Huang et al. [49]	2022	Q1	Fast R-CN, GoogLeNet, SURF	Private	Accuracy	90%
Alshayeji et al. [47]	2023	Q1	SVM	Private dataset	Sensitivity, Precision and AUC	97.81%, 97.9% and 0.9995
Rania et al. [50]	2020	C	U-Net	ESCALE	Accuracy, IoU and DSC	94.96%,94.86% and 97.25%
Bougrine et al. [60]	2019	B1	FCN, SegNet, U-Net	Private dataset	Dice Similarity Coefficient (DSC), standard deviations (STD)	(FCN = 96.16% ± 0.85%) (SegNet = 97.26% ± 0.69%) (U-Net = 74.35% ± 9.58%)
Wang et al. [119]	2020	Q1	MobileNetV2 and CCL	Private dataset consisting of 1109 images	Precision, Recall, and the Dice coefficient	91.01%, 89.97% and 90.47%
Lan et al. [120]	2023	Q1	FusionSegNet	Private dataset	AUC, Accuracy, Sensitivity, Specificity, F1-score	98.93%, 95.78%, 94.27%, 96.88%, 94.91%
Jishnu et al. [121]	2023	Not Yet Assigned	AFSegGAN	DFUC2021	Dice score, IoU	93.11%, 99.07%
Jiao et al. [122]	2025	Q1	UFOS-Net with EMS and MODA	DFU Segmentation	Dice, IoU	77.45%, 66.64%
Niri et al. [123]	2025	Q1	Dual Attention U-Net with SE Blocks	Wound Segmentation	Dice, IoU	94.1%, 89.3%

**Table 10:** Characteristics of classification-targeted DFU studies including author, year, dataset employed, used ML model, validation metrics, and their results.

Author [Refs.]	Year	Journal Rank (SJR)/Conference Rank (Qualis)	ML/DL Model	Dataset	Validation Parameter	Value
Botros et al. [124]	2016	Not Yet Assigned	SVM with a global average pooling (GAP)	Private dataset	Accuracy and Precision	96.4% and 96.4%
Kasbekar et al. [125]	2017	Q1	Decision tree	Private dataset	Error rate and Accuracy	3.6% and 94%
Adam et al. [126]	2018	Q2	SVM, Discrete Wavelet Transform (DWT) and Higher Order Spectra (HOS)	Thermograms images 33 healthy and 33 with type 2 diabetes	Accuracy, Sensitivity and Specificity	89.39%,81.81% and 96.97%
Goyal et al. [100]	2018	Q1	CNN (DFUNet) and Conventional ML(CML)	DFU A(I)	Sensitivity, F-measure, Specificity, Precision and AUC	0.929, 0.931, 0.908, 0.942 and 0.950
Vardasca et al. [127]	2018	Q3	SVM and K-NN	Private dataset	Accuracy and Positive prediction	92.5% and 20%
Goyal et al. [63]	2018	Q1	Faster R-CNN, MobileNet, InceptionV2	1775 foot images with DFU	mAP and Speed	91.8% and 48 ms
Vardasca et al. [128]	2019	Q3	ANN, SVM and k-NN	Private	Accuracy, Specificity and Sensitivity	81.25%, 80 and 100%
Gamage et al. [73]	2019	B1	Pre-trained CNN, ANN, RF, SVM and Singular Value Decomposition (SVD)	A private dataset has 2400 images	Accuracy and F-score	96.22% and 0.9610
Alzubaidi et al. [129]	2020	Q1	QUTNet based on D-CNN, KNN and SVM	DFU (alzubaidi)	Precision, Recall and DSC	95.4%, 93.6%, 94.5%
Goyal et al. [68]	2020	Q1	Faster R-CNN and Superpixel Color Descriptor	DFU B (II)	Accuracy in ischemia and infection classification	90% and 73%
Cruz et al. [81]	2020	Q1	DFTNet	PLANTAR THERMO-GRAM	Sensitivity, Specificity and Accuracy	0.95,0.94 and 0.94
Amin et al. [59]	2020	Q1	YOLOv2-DFU	Part (B)(II)	Sensitivity, Recall and Precision and Accuracy	0.99 and 0.97 accuracy on infection and ischemia, 0.98 and 0.97 IOU on ischemia and infection
Liu et al. [65]	2020	Not Yet Assigned	Faster R-CNN	Private	Accuracy	95%
Padierna et al. [78]	2021	Q2	SVM	Private	Accuracy, Sensitivity and Specificity	92.64, 91.80 and 93.59
Niri et al. [62]	2021	A1	Spx-based FCNs	Private and ESCALE	Accuracy, Sensitivity, Specificity, Precision, and DSC	92.68%, 74.53%, 94.39%78.07% and 75.74%
Cassidy et al. [66]	2021	Q3	Faster R-CNN, FRCNN ResNet101, FRCNN Inception-v2-ResNet101, YOLOv5 and EfficientDet	DFUC 2020	Recall, Precision, F1 score and mAP	F1 scores= 0.6784, 0.6623, 0.6716, 0.6612 and 0.6929
Galdran et al. [130]	2021	Not Yet Assigned	Big Image Transfer (BiT),EfficientNet, Vision Transformers (ViT), Data-efficient Image Transformers (DeIT)	DFUC2021	DSC, AUC, Recall and Precision	62.16, 88.55, 65.22 and 61.40
Selle et al. [131]	2021	Not Yet Assigned	SVM	Private	Accuracy	96.42%
Xu et al. [72]	2021	Q1	A pre-trained vision transformer models class knowledge banks(CKBs)	DFU B(II)	Accuracy, Sensitivity, Precision, Specificity, DSC and AUC score	90.90, 86.09, 95, 95.59, 90.30 and 96.80
Da et al. [67]	2021	B	Faster R-CNN	DFUC 2020	mAP and DSC	91.4 and 94.8
Alzubaidi et al. [129]	2021	Q1	DFU_QUTNet and SVM	DFU (alzubaidi)	Precision, Recall and DSC	95.4%,93.6% and 94.5
Yap et al. [69]	2021	A	EfficientNetB0 with data augmentation and transfer learning	DFUC2021	Average Precision, Recall and F1-Score	0.57, 0.62 and 0.55
Bloch et al. [77]	2021	A1	EfficientNet	DFUC2021	DSC	60.77%
Khandakar et al. [80]	2021	Q1	MobilenetV2	PLANTAR THERMO-GRAM	DSC	97
Das et al. [132]	2022	Q2	ResKNet	DFU B(II)	AUC	0.99 for ischemia and AUC=0.89 for infection
Al-Garaawi et al. [70]	2022	Q1	DFU-RGB-TEX-NET	DFU A(I) and DFU B(II)	AUC and DSC	0.981%, 0.952% on Part-A and 0.820%, 0.744% on Part-B infection
Al-Garaawi et al. [71]	2022	Q3	GoogLNet CNN	DFU A(I) and DFU B(II)	Sensitivity, Specificity, Precision, Accuracy, DSC and AUC	0.93, 0.900.94, 0.92, 0.93 and 0.97
Husers et al. [75]	2022	Not Yet Assigned	MobileNetV1	Private dataset	Accuracy, Precision, Recall and F1-score	69%, 67%, 69% and 0.73
Santos et al. [76]	2022	B1	VGG-16, VGG-19, Resnet-50, InceptionV3, and Densenet-201	Private	Accuracy and Kappa index	95.04% and 91.85%
Yogapriya et al. [133]	2022	Q2	DFINET	DFU B(II)	Accuracy and and MCC	91.98% and 0.84
Jain et al. [79]	2022	PhD Thesis	SIFT and SURF combined with BOF, and SVM	PLANTAR THERMO-GRAM	Accuracy, Specificity and Sensitivity	91.23%, 91.50% and 92.41%
Jain et al. [134]	2022	Not Yet Assigned	ProNet, AlexNet, ResNet	PLANTAR THERMO-GRAM	Accuracy, Precision, Sensitivity, Specificity and F1-Score	98.9%, 1.000, 0.978, 1.000 and 0.988
Khandakar et al. [135]	2022	Q1	MLP Classifier, XGBoost Feature Selection and choosing Top 2 Features	PLANTAR THERMO-GRAM	Accuracy, Precision, Sensitivity, DSC and Specificity	0.91, 0.91, 0.91, 0.91, 0.91 and 95.83
Khandakar et al. [82]	2022	Q1	VGG 19 CNN	PLANTAR THERMO-GRAM	Accuracy, Precision, Sensitivity, F1-Score and Specificity	94.76, 94.89, 94.67, 94.73 and 97.32
Munadi et al. [84]	2022	Q2	ShuffleNet and MobileNetV2	PLANTAR THERMO-GRAM	Accuracy Sensitivity, Specificity, Precision and F-Measure	1.0, 1.0, 1.0, 1.0 and 1.0
Anaya et al. [85]	2022	Q1	ResNet50v2	PLANTAR THERMO-GRAM	Accuracy, Sensitivity, Specificity and FPR	100%, 100%, 100% and 0%
Balasenthi- lkumaran et al. [87]	2022	Q4	ANN, QSVM, linear discriminant, logistic regression and Gaussian naïve Bayes	Private	Accuracy and F1 score	93.3% and 0.95
Filipe et al. [83]	2022	Q2	Logistic Regression, SVM Quadratic, Linear SVM, 3-NN and weighted k-NN	PLANTAR THERMO-GRAM	Accuracy, Sensitivity, Specificity, Precision, AUC and F-Score	0.924, 0.833, 0.958, 0.882 and 0.857
khosa et al. [136]	2023	Q2	Custom Model	PLANTAR THERMO-GRAM	Sensitivity, Specificity, Accuracy, F1-Score, AUC	0.97, 0.958, 0.97, 0.891, 0.976
Reyes et al. [24]	2023	Q1	DFU_VIRNet	DFU (alzubaidi)	AUC, F-score	0.9982 and 0.9928 for ischemia and 0.9121, 0.8363 for infection
Nagaraju et al. [137]	2023	Q1	Inception-ResNet-v2	DFU (alzubaidi)	Accuracy	99.29
Biswas et al. [138]	2023	Q1	DFU_MultiNet	DFU (alzubaidi)	Accuracy	99.06
Toofanee et al. [139]	2023	Q1	DFU-SIAM	DFU2021	macro-F1 score, F1-score	0.623, 0.549 for ischemia and 0.628 for infection
Das et al. [140]	2024	Q1	HCNNet	DFU-Part(B)	AUC	0.999
Fadhel et al. [141]	2024	Q2	DFU_FNet and DFU_TFNet	Real-time	Accuracy, Precision, F1-Score	99.81%, 99.38% and 99.25%
Patel et al. [142]	2024	Q1	Multi-modal deep learning framework	AZH, Medetec	Accuracy	74.79–100%
Almufadi et al. [143]	2025	Q2	E-DFu-Net	Transfer learning	Accuracy (Ischemia, Infection)	97%, 92%
Ajay et al. [144]	2025	Q1	Dense-ShuffleGCANet	Attention-driven mechanisms	Robustness	Strong across diverse datasets
Karthik et al. [145]	2025	Q1	Swin Transformer + Multi-scale Attention	DFUC-2021	F1-Score	80%
Ullah et al. [146]	2025	Q1	Eff-ReLU-Net	EfficientNet-B0 + ReLU	Accuracy (Medetec, AZH)	92.33%, 90%
Reis et al. [147]	2025	Q1	CNN Fairness Evaluation	VGG16, VGG19, MobileNetV2	Disparities in Skin Tone Performance	Highlighted need for inclusivity
Maurya et al. [148]	2025	Q1	MCTFWC (CNN-Transformer)	Medetec, AZH	Accuracy	High across wound types
Fitriah et al. [149]	2025	Q2	MobileNetV2-based DFU Severity Classification	Low-resource settings	Efficiency	Strong results for severity grading
Bansal et al. [150]	2024	Q3	ML Classifiers + Multivariate Features	Custom dataset	Accuracy	Promising but limited
Karthik et al. [144]	2024	Q1	Dense-ShuffleGCANet	Attention mechanisms	Robustness	Strong performance

**Table 11:** Characteristics of hybrid Classification and Segmentation-targeted DFU studies including author, year, dataset employed, used ML model, validation metrics, and their results.

Author [Refs.]	Year	Journal Rank (SJR)/Conference Rank (Qualis)	ML/DL Model	Dataset	Validation Parameter	Value
Wannous et al. [93]	2010	Q1	SVM and Mean Shift iterative color clustering algorithm	850 color images	Overlap score, Sensitivity, Specificity, Success rate, Accuracy	73.8%, 77%, 92%, 84%, 88%
Mukherjee et al. [90]	2014	Q2	SVM and Bayesian classifier, color conversion and fuzzy divergence for segmentation	total = 767 images where granulation tissue = 222, slough tissue = 451 and necrotic tissue = 94	Accuracy	86.94%, 90.47%, and 75.53%, for classifying granulation, slough, and necrotic tissues, respectively
Babu et al. [92]	2018	Not Yet Assigned	Naive bayes and Hoeffding tree classifier and Particle Swarm Optimization (PSO)	3 DFU images used to test the method	Accuracy, Sensitivity and Specificity	90.90%, 100%, 87.5% by Naïve Bayes and 81.81%, 100%, 77.7% by Hoeffding Tree
Godeiro et al. [91]	2018	B2	For classification SegNet, where segmentation U-Net	30 color image foot and hand	Accuracy, Specificity, Sensitivity, and DSC	0.9610, 0.9876, 0.9128 and 0.9425
Wijesinghe et al. [88]	2019	Not Yet Assigned	R-CNN and D-CNN Module	400 DFU images	SUS	88.5
Maldonado et al. [89]	2020	Q2	Pretrained Mask R-CNN and Gaussian distribution	Private-DB1 had a total of 108 images where DB2 contained a total of 141 images	Accuracy	90.28%
Zhou et al. [86]	2025	Q1	Mask2Former, Deeplabv3Plus, Swin-Transformer	DFU Dataset (671 images)	Accuracy, mIoU	91.85%, 65.79%

## Data Availability

The data used in this study has been provided in the references of this paper and Supplementary File. PRISMA 2020 Checklist available at: PRISMA checklist, Supplementary File S6.

## Supplementary Materials

The following supporting information can be downloaded at: https://www.mdpi.com/article/10.3390/biomedicines13122928/s1, S1: Advances in Image-Based Diagnosis of Diabetic Foot UlcersUsing Deep Learning and Machine Learning: A Systematicn Review (Supplementary File); S2: Sheet “Included & excluded studies”; S3: Sheet “Features” Feature-related extractions; S4: QUADAS-2 Quality Assessment Analysis; S5: Quality assessment using GRADE. S6: PRISMA 2020 Checklist. S7: Inplasy protocol 2022110128.

## Abbreviations

AI	Artificial Intelligence
AUC	Area Under Curve
CNN	Convolutional Neural Network
CT	Computed Tomography
DCNN	Deep Convolutional Neural Network
DFU	Diabetic Foot Ulcer
DL	Deep Learning
DM	Diabetes Mellitus
DSC	Dice Similarity Coefficient
FPR	False Positive Rate
mAP	mean Average Precision
MCC	Matthews Correlation Coefficient
ML	Machine Learning
MRI	Magnetic Resonance Imaging
NN	Neural Network
PAD	Peripheral Arterial Disease
QUADAS-2	Quality Assessment of Diagnostic Accuracy Studies-2
RGB	Red, Green, Blue
RMSE	Root Mean Square Error
ROC	Receiver Operating Characteristic curve
SUS	System Usability Scale
SVM	Support Vector Machine
YOLO	You Only Look Once
