# Supplementary material for: Advances in Image-Based Diagnosis of Diabetic Foot Ulcers Using Deep Learning and Machine Learning: A Systematic Review
Source: Biomedicines. 2025 Nov 28;13(12):2928. doi: 10.3390/biomedicines13122928 (PMC12730623; doi:10.3390/biomedicines13122928)
Supplement: Supplementary file 1 [file biomedicines-13-02928-s001.zip › S1_DFU_Systematic_review__Supplementary_file_.pdf]

biomedicines

Article

Advances in Image-Based Diagnosis of Diabetic Foot Ulcers Using Deep Learning and Machine Learning: A Systematic Review (Supplementary File)

Haifa F. Alhasson [1]* and Shuaa S. Alharbi [1]

[1] Department of Information Technology, College of Computer, Qassim University, Buraydah 52571, Saudi Arabia; E-mail: hhson@qu.edu.sa (Haifa F. Alhasson), shuaa.s.alharbi@qu.edu.sa (Shuaa S. Alharbi)
* Correspondence: Haifa F. Alhasson, Email: hhson@qu.edu.sa

check for updates

Received:
Accepted:
Published:

Citation: Alhasson, H.; Alharbi, S. Diabetic Foot Ulcer Diagnosis. *Biomedicines* **2024**, *1*, 0. https://inplasy.com/inplasy-2022-11-0128/

Copyright: © 2024 by the authors. Licensee MDPI, Basel, Switzerland. This article is an open access article distributed under the terms and conditions of the Creative Commons Attribution (CC BY) license (https://creativecommons.org/licenses/by/4.0/).

1. ML/DL models Used in Diabetic Foot Detection, Segmentation, and Classification

One of the earliest AI experimental trials to detect and segment DFU was Wang *et al.* 2016[1,2] where images from a capture box were used to perform wound image assessment algorithms to calculate the overall wound area, colour segmented wound areas, and a and calculate healing score, to provide a quantitative assessment of the wound healing status using support vector machines (SVM) to determine the wound boundaries on foot ulcer images. As a result of physical contact between wounds and capture boxes, this method exhibited several limitations, such as infection risk. Later, the work of Goyal *et al.* [3–6] trained different models capable of classification, detection, and segmentation.

1.1. Detection Task

1.1.1. Colour Image-based Approaches

Han *et al.* [7] introduced the real-time detection and location method for Wagner grades of DFU based on refinements of YOLOv3. Similarly, Brungel *et al.* [8] investigate Detection Transformer (DETR) as a representative of novel transformer-based architectures, YOLOv5 [9], matured updated versions of [10] and the original YOLOv4 that is targeted at mobile devices. The two approaches are compared on the DFU2021 dataset for detecting DFUs using typical images for wound care documentation. The mobile focus and decent performance of YOLOv5 make it suitable for performing DFU detection tasks directly on mobile devices. In Cassidy *et al.* [11], an analysis of the automatic detection of DFUs is performed using a smartphone and cloud-based framework. By using TypeScript code, the system enables the deployment of mobile apps to multiple platforms.

Using EfficientNet, Thotad *et al.* [12] proposed an early detection and prognosis for diabetic foot ulcers. The EfficientNet algorithm was applied to 844 images of feet containing healthy and diabetic ulcerated feet. The performance of the system is improved by carefully balancing the width, depth, and resolution of the network, compared to earlier models using EfficientNet. Sarmun *et al.* [13] combined deep learning models to improve excretion in the context of diabetic foot ulcer recognition and reducing the number of false-positive infections. And it is a powerful theoretical approach to pursuing in the clinical dataset. Sendilraj *et al.* [14] announced the development of a deep learning-based platform named DFUCare, which is used for diabetic foot ulcer monitoring as well as clinical examination. Biswas *et al.* [15] have used a multi-scale feature exudation method to develop an explainable AI-based approach dubbed XAI-FusionNet in order to diagnose without losing transparency. According to El-Kady *et al.* [16], an 85 percent rate in clinical

examination is good. This technique has been created by integrating the ResNet and GAN architectures. Azeem *et al.* [17] mentioned that their concept for this program appears to be too reasonable. Techniques based on the SSD and YOLO designs were used to optimize the strategies. These strategies were tried at the 2024 IEEE HONET Conference. Verma [18] demonstrated the promise of intelligent image processing methods for early ulcer detection by employing a strong deep learning network in their research. Busaranuvong *et al.* [19] launched Guided ConDiff to integrate diffusion models into medical into diabetic medical ulters to achieve better infection detection. This study showed the ability to utilize a robust deep neural network with DL. Eldin *et al.* [20] found that deep neural networks have significant benefits for early detection of diabetic foot ulcers to enhance diagnostic efficacy. Rathore *et al.* [21] applied a feature-based uncertainty model for early detection and incorporation of most prevention avoidances in ulcer identification was referred. Masquerading is used by Debnath *et al.* [22] as absurd AI to detect research and positively use materials in recognition of early diagnosis. With Grad-CAM and LIME DFU_DIALNet, Mahmud *et al.* [23] think companies can generate easily verifiable viable and low-priced on the basis of ideas and can allow vilification. Girmaw *et al.* [24] optimized primary identification of enhance MobileNetV2, which would be needful for internal restorative potential replication. CNN and SMOTE-IPF mechanisms discussed by Pradhana *et al.* [25] will help increase the speed of diagnosis in the discovery and treatment of diabetes using thermogram images. This shows that research examination among inattention samples should be promising for tackling possible problems.

1.1.2. Thermal/Thermographic Image-based Approaches

Nag *et al.* [26] rely on finding abnormal points in thermal images of diabetic patients and then segmenting the images based on the abnormal points. After segmenting the images, they trained three different classifiers: Support Vector Machines (SVM), k-Nearest Neighbors (k-NN), and Decision Trees (DT).

1.1.3. Hyperspectral Image-based Approaches

Using emerging photonics-based technology, and definitive physiological characteristics, Dremin*et al.* [27] worked on the first in vivo human clinical study to report a label-free, non-invasive polarization-sensitive approach to investigate DM skin complications based on using ANN. The system utilized polarization-based hyperspectral imaging to detect DM skin complications at a very early stage. Lindberg *et al.* [28] demonstrate that this type of image can be used with a variety of techniques other than DL or ML.

1.2. Segmentation Task

A key component of the care and diagnosis protocol is the segmentation of wound boundaries in images in order to approximate wound size and provide quantitative measurements for the treatment [29].

1.2.1. Colour Image-based Approaches

Diabetes skin lesions are heterogeneous, and digital camera images contain a lot of noise and variation of lighting, making wound extraction difficult. The authors in [1] present a novel superpixel segmentation technique followed by a cascaded two-stage SVM classifier for identifying wound boundaries in foot ulcer images. This scheme controls the lighting and range.

In 2019, with the advancement of CNN use, a DL-based method was proposed based on CNN (Cui *et al.* [30]) to accurately segment wound regions, generating a probability map. Using these probability maps, the wound region was extracted. Additionally, the researchers in [31] examined whether CNN could segment wounds of DFUs and venous leg

ulcers using sacral pressure ulcer (PU) data sets, and which CNN architecture was better for segmenting wounds. In this study, CNNs were used with four different types of architectural structures: (SegNet, LinkNet, U-Net [32] and U-Net with the VGG16 [33] Encoder Pre-Trained on ImageNet (Unet_VGG16)). Of the four the U-Net architecture produced the best results. Later, Gamage *et al.* [34] introduced Mask-RCNN-based Segmentation employing a data augmentation technique. The two backbone options for Mask-RCNN [35] are provided as a comparison of their performance (ResNet-50 and ResNet-101). They applied the two versions of their model to a private dataset consisting of 2400 RGB images compared with U-Net.

Wang *et al.* [36] used MobileNetV2 [37] and connected component labelling (CCL) to build up a novel convolutional framework that segments wound regions from natural images. This model is well-known for its lightweight and lower computation-burden architecture. The framework's performance is comparable to deeper NNs. Later, Jawahar *et al.* [38] compared mask-based segmentation, colour space-based segmentation, and K-Means clustering as methods for segmenting DFUs. As compared to the other two methods, K-Means clustering provided better-segmented regions. Rania *et al.* [39] investigate which DL approaches are suitable to be applied to a small database and are feasible to get accurate segmentation. They tested three different fully convolutional networks and conclude that U-Net is the best choice for that target with a Dice Similarity Coefficient of 97.25% and an intersection over union index of 94.86%. Munoz *et al.* [40] used Mask R-CNN and the transfer learning concept to locate the ulcer delineation region automatically.

While DL-based methods for automatically segmenting wounds have shown promising results, they require large datasets for training and it is unclear which method performs best. Currently, Wang *et al.* [29] proposed the Foot Ulcer Segmentation challenge (FUSeg) organized in conjunction with MICCAI 2021. The challenge was based on an image dataset containing 1, 210 pixel-wise annotated foot ulcer images collected from 889 patients and split into a training set with 1010 images and a testing set with 200 images for evaluation.

The winners in this challenge were: Mahbod *et al.* [41], Huang *et al.* [42] and Galdran *et al.* [43]. Mahbod *et al.* [41] used U-Net and LinkNet [44]. Instead of using the models in their plain forms, pre-trained CNNs were used in the decoder parts of the models. For LinkNet, a pre-trained EfficientNetB1 model [45] was used, and the EfficientNetB2 model [45] was utilized for U-Net. Hernandez *et al.* [46] presented a model for automatic segmentation based on the U-Net architecture to identify and delineate images of feet using multimodal imagery. Huang *et al.* [42] proposed the Harmonic Densely Connected Network (HarDNet-MSEG). HarDNet-68 was adopted as the encoder. It consists of repeated application of HarDNet blocks, batch normalizations (BN), and max pooling layers. The arrangement of the layers follows the standard Conv-BN-ReLU order to enable the folding of BN, then, the decoder is a cascaded partial decoder [47]. This technique allows the shallow features to be discarded and allows for more computing on the deeper layers' features.

Galdran *et al.* [43] proposed a semantic segmentation based on the sequential use of two encoder-decoder segmentation networks where the second network receives as input the concatenation of the prediction from the first one. Chang [48] proposed a region-based method that uses superpixels to assist. DL models (U-Net, DeeplabV3 [49], PsPNet [115], FPN [50,51], and Mask R-CNN) were trained on two large private datasets with an encoder (ResNet-101). The highest performance was achieved by DeeplabV3. Chitra [52] performed wound image segmentation and tissue classification by grouping with the Random Forest (RF) algorithm [53].

During the MICCAI Diabetic Foot Ulcer Segmentation Challenge (DFUC2022), in 2021 Liao *et al.* [54] produced a new state-of-the-art CNN called HarDNet-DFUS by enhancing the backbone and replacing the decoder of HarDNet-MSEG[42], which was a SOTA for

polyp segmentation. HarDNet-DFUS was trained using the DFUC2022 dataset [55] for DFUC2022. Most Recently, Huang *et al.* [56] built a framework to perform a qualitative evaluation of DFU patients. They used the Fast R-CNN network and transfer learning for object recognition, and then employed other techniques like Speeded Up Robust Features (SURF) for Feature detection to analyze the classification, location, and size of wounds. Moreover, Heras *et al.* [57] proposed a segmentation process that involves two steps: the segmentation of ulcer regions and the post-processing of the segmentation results. After comparing five ML models, a trained ML model was selected to classify pixels within the region of the ulcer.

Recently, In order to identify DFU images from foot ulcer images, Lan *et al.* [58] proposed combining global foot features with local wound features (FusionSegNet). To segment foot ulcer wounds, the authors apply a wound segmentation module, which instructs the network to pay attention to the area surrounding the wound. Jishnu *et al.* [59] aimed to design a wound management system called AFSegGAN, which estimates the wound morphological characteristics and uses a conditional generative adversarial network (cGAN) to automate foot ulcer segmentation. Furthermore, they balanced the GAN network training and enhanced the segmentation performance by employing adversarial loss and patch-level comparison of the discriminator network.

Recently, Jiao *et al.* [60] ntroduced UFOS-Net: a new kind of segment representation model with Enhanced Multi-scale Segmentation (EMS) module and MODA data enhancements specifically for diabetic foot ulcer (DFU) segmentation. Their model reached a Dice coefficient of 0.7745, which far outclassed previous methods in segmentation performance. It showed better separability than existing methods on the SRRSH-DF and DFUC2022 datasets. Niri *et al.* [61] developed dual attention U-Net model, blending attention gates from the original U-Net and squeeze-and-excitation blocks for more precise wound segmentation. Their study achieved 94.1% Dice coefficient, and 89.3% IOU demonstrating that integrating dual attention mechanisms with the transfer learning possible but optimal.

1.2.2. Thermal/Thermographic Image-based Approaches

Many studies prove that Infrared thermography is a promising modality for such a system. The purpose of the study by Kaabouch *et al.* [62] was to explore whether thermal imaging can be used to assess the integrity of the skin and its various layers. A genetic algorithm and an asymmetry analysis are used to analyze infrared images to perform early detection of foot ulcers. This study proved the efficiency and reliability of using Thermal images.

Using a U-Net-based model, Bouallal *et al.* [63] segmented thermal images. A U-Net was trained by combining the thermal and colour images from the FLIR ONE Prothermal camera. Compared to a thermal-only approach, they concluded that the multimodal approach performed better. Based on these results, thermal camera temperature assessment could be useful in the diagnosis of diabetic foot problems. Recently, Bouallal *et al.* [64] developed an automated, robust, and accurate way to segment diabetic feet. To accomplish this, they used a deep NNs architecture based on the encoder-decoder concept known as Double Encoder-ResUnet (DE-ResUnet). A residual network and a U-Net architecture were combined in this network, which also used RGB (Red, Green, Blue) colour channels fused with thermal information to enhance segmentation accuracy.

Based on the Snake energy function, Bougrine *et al.* [65] incorporated prior shape information, namely the atlas of the plantar foot contour, as an extra term. This term can be calculated as a curvature difference between the Snake curve and the atlas curve of the plantar foot surface. Later, they extended their work [66] and used NNs to segment plantar foot thermal images taken by a smartphone camera. There were three different DL

methods compared: Fully Convolutional Networks (FCN) [67], SegNet [68], U-Net [32], and a previously developed contour-based method. In their database, 80% of the data is used for training and 20% for testing the three DL networks. The results proved that the SegNet method significantly achieved better results than the other three methods with a Dice Similarity Coefficient (DSC) equal to 97.26%.

Most recently, by using the plantar thermogram database, Alshayeji *et al.* [69] were able to detect an increase in plantar temperature and diagnose it early. Unlike other methods, this method is less sensitive to environmental conditions, such as smoke and weather. Combining the Scale Invariant Feature Transform (SIFT) and SURF methods with the Bag of Features (BOF) technique, they extracted features that are invariant to translation, rotation, and scaling transformations. To compare ML models trained with DL and handcrafted features, they used different pre-trained DL networks. To binary classify normals from DFUs, conventional ML classifiers were preferred. Based on SURF-BOF training of an SVM [70], the proposed model achieved 97.81% classification accuracy, 97.9% precision, and 0.9995 Area under the ROC curve (AUC).

1.3. Classification Task

Electronic noses (e-noses) have been found to be effective in early studies evaluating DFU classification. The use of e-noses for identifying bacteria on DFUs has been found to be effective in some studies. Abdullah *et al.* [71] proposed an electronic nose for detecting bacteria in diabetic foot ulcers. This data was analyzed using Principal Component Analysis (PCA) and Linear Discriminant Analysis (LDA). In another work, Yusuf *et al.* [72] evaluated E-nose performance in vitro to identify the causative bacteria for a diabetic foot infection. The evaluation showed that the e-nose was able to identify and differentiate between single and poly microbial cultures that had been isolated from DFUs and cultured on agar media. Several statistical approaches were employed, including SVM, k-NN, LDA and Probability Neural Network (PNN) [73].

Treatment and referral decisions may be guided by accurate stratification of wounds according to their likelihood of slow healing. Based on the information collected, Jung *et al.* [74] developed a predictive model for delayed wound healing using modern ML methods and feature engineering. By randomly assigning patients' information to training and test sets, wounds were divided into training and test sets. They compared three different techniques: L1 regularized logistic regression (lasso), RF, and gradient-boosted tree models. They concluded that the best single model was a gradient-boosted tree model.

1.3.1. Colour Image-based Approaches

Botros *et al.* [75] performed a DFU prediction based on dynamic pressure distribution. With newly extracted features, ML techniques, and SVM, they were able to achieve 94.6% in classification accuracy and 95.2% in precision. With newly extracted features, ML techniques, and SVM, they were able to achieve 94.6% in classification accuracy and 95.2% in precision.

A classification of DFU by severity stage is essential for treatment planning and diagnostics. Many studies like Gamage *et al.* [76] have constructed a CNN based on DenseNet201 architecture and combined it with a global average pooling (GAP) layer to predict six classes of severity stages. This practice also includes optimizing processing time and memory usage. The proposed architecture could achieve an accuracy of over 96%.

Patel *et al.* [77] evaluated the need for a more effective, easier way to diagnose DFUs to enable patient healing at an earlier stage. They analyzed the most popular ML classification techniques used in medical imaging. To classify wound regions, Bayesian networks, NNs, k-NN classifiers, fuzzy logic techniques, and SVMs were employed.

In order to estimate the risk of amputation among diabetic patients, various classification systems have been proposed *e.g.*, Kasbekar *et al.* [78] used the decision tree algorithm on data collected from 301 diabetic foot patients. The results showed that the most influential features are Doppler flow measurements and the Wagner grading of ulceration. Liu *et al.* [79] aimed to develop a Faster R-CNN-based Wagner level classification detection model for diabetic feet. A professional team of doctors performed the annotation and inspection of the data set to ensure the validity of the data.

An extensive database of 1775 images of the DFU was built up and annotated by Goyal *et al.* [5]. Using an annotator, they were able to outline the region of interest of the DFU. Using five-fold cross-validation, the faster R-CNN [80] with InceptionV2 model achieved an overall average precision of 91.8%, a speed of 48 ms for inferencing one image, and an extremely small model size. Then they extended their study in [4] to propose the use of ML algorithms to extract the features of DFU and healthy skin patches to understand the differences. An innovative CNN architecture, DFUNet, identifies the differences between healthy skin and DFU by better feature extraction. A cross-validation study of 10 folds found that DFUNet had an AUC score of 0.961, which was superior to both traditional ML and DL classifiers.

Current research focuses on ischaemia and infection recognition. Goyal *et al.* [6] extend their work to distinguish between ischaemia and infection in DFU. *Ischaemia* is the insufficient supply of blood that may impair the healing of DFUs. A diagnosis of ischaemia can be made by palpating the pulses in the feet and by measuring the blood pressure in the toes and feet. In contrast the term infection refers to a bacterial infection in soft tissue or bone in the DFU, which is defined as three or more classic signs of inflammation or purulence. For the better identification of ischaemia and infection in DFU, the Ensemble CNN model [81] was used in [6]. It incorporates the bottleneck features from multiple CNN models (Inception-V3, ResNet50, and InceptionResNetV2) and uses an SVM classifier to provide a prediction. It is an extremely effective CNN approach to obtaining very good accuracy on challenging datasets. Their model is supported by a natural data-augmentation method, which identifies the region of interest in foot images and focuses on the salient features there. Moreover, 16 layers of CNNs were proposed by Amin *et al.* [82], including the following: one input, three convolutions, three batch-normalizations, one average pooling, one skip convolutional, one ReLU, one addition (element-wise addition of two inputs), fully connected, softmax and classification output layers for classification, and YOLOv2-DFU for localization of infection/ischaemia models.

After the DFU Grand Challenge (DFUC) 2020, Cassidy *et al.* [83],, the methods tested included Faster-RCNN and Inception-v2-ResNet101, FRCNN Inception-v2-ResNet101, YOLOv5, and EfficientDet [84]. Observations have shown that these networks provide promising results.

Adding more layers to a typical CNN does not improve its performance, rather it causes it to perform worse due to the gradient involved. Considering this, Alzubaidi *et al.* [85] designed a CNN based on the idea of increasing the width of the network while keeping the depth of the network the same. A novel deep CNN, DFU_QUTNet, is proposed for the automatic classification of the normal skin (healthy skin) class versus the abnormal skin (DFU) class. The authors compared three pre-trained DL networks for the same task (GoogleNet, VGG16, and AlexNet) that had been fine-tuned, re-trained, and tested. Using data augmentation techniques and changes to parameter settings, their proposal in Da *et al.* [86] is to improve the original Faster R-CNN algorithm. The researchers used a training dataset consisting of 2000 images from the DFUs 2020 challenge. The training was validated using Monte Carlo cross-validation, achieving 91.4% precision and 94.8% F1 score. In addition, based on comprehensive experiments performed by [87], it appears that

modern CNNs are still capable of outperforming Transformers in low-data regimes, likely as a result of their ability to better exploit spatial correlations. A recent Sharpness-Aware Minimization (SAM) optimization algorithm has also been demonstrated to improve the generalization capability of both types of models considerably.

As DL models are tested, they make predictions based on an individual input image and trained parameters, without explicitly taking into account the knowledge in the training data. To make better use of the knowledge that can be extracted and represented by class knowledge banks (CKBs), Xu *et al.* [88] proposed the use of trainable units that can be employed to extract and represent knowledge from training data. As each unit in a CKB performs similarity calculations with an image representation, it is used to calculate the similarity between those images.

Recent advances have been made in the classification of normal and abnormal DFU skin. DFU_SPNet [89] is presented as a unique stacked parallel convolutional layer-based network. It is comprised of three blocks of parallel convolution layers, each with a different kernel size for the abstraction of local features and global features, respectively. The proposed DFU_SPNet, trained using SGD (with momentum) optimizer, outperformed the current state-of-the-art results.

Data preparation and collection of DFUC2021 are described by Yap *et al.* [90], as well as data analysis and ground truth annotation. A UMAP projection is used to analyze the separability of the dataset based on image hashing. Five key backbones of DL are benchmarked, namely VGG16, ResNet101, InceptionV3, DenseNet121 and EfficientNet [84] on DFUC2021. According to their observations, Efficient-NetB0 with data augmentation and transfer learning provides the best results classified to multi-class (4-class) classification. Later, combination of Faster-RCNN [80], YOLOv5, and EfficientDet [84] was used to identify DFUs in Yap *et al.* [91]. With faster R-CNN, false positives are reduced, and detection performance is enhanced.

Several classification learning algorithms have been observed to have low predictive accuracy when applied to classes that occur infrequently. To overcome severe class imbalances, Bloch *et al.* [92] used EfficientNet with an extension strategy involving pseudo-labeling for unlabeled images and extensive use of synthetic images. As a result, the extended training dataset is 8.68 times larger than the baseline and shows a ratio of real-to-synthetic images of 1 : 3.

The most recent focus is aiming to provide more accurate diagnosis and a less-subjective decision during DFU examination by skin telemonitoring. Niri *et al.* [93], developed a smartphone-based system for skin telemonitoring that can be used during the examination of DFU tissues for support of medical diagnoses and decisions, encompassing automatic ulcer segmentation and tissue classification. Lopez *et al.* [94] developed automatic classification techniques for DFU images and examined various approaches to traditional computer vision methods and feature extraction from a CNN. The CNN Densenet201 was used to extract features for an SVM classifier. Based on this, the proposed alternative of combining a pre-trained CNN model with automatic classifiers shows promising results.

Interestingly, researchers have shifted their focus in recent years to combining handcrafted features with deep features, resulting in an excellent performance in several computer vision tasks. Recently, RGB-based deep models for classification (Al-Garaawi *et al.* [95]) showed that feeding an additional feature (texture information) to the CNN model provides additional benefit to the standard RGB-based deep models, and even better performance can be achieved by combining RGB images and texture features as inputs tothe CNN. Moreover, Alg *et al.* [96] proposed a deep CNN-based system consisting of three stages: feature extraction, feature fusion, and DFU classification. The feature fusion is

performed by concatenating all feature vectors into a single vector. Then, an RF classifier (RF) is performed on the fusion vectors. This technique provides enhanced performance in DFU, ischaemia, and infection classification.

The improvement of external validity of the existing models in literature has been the most recent trend to avoid overfitting certain data. The improvement can be done by employing adaptive NNs, augmentation, batch normalization, and dropout operations. Using the MobileNetV1 model, Husers *et al.* [97] developed a CNN model for image classification. Among the advantages of MobileNetV1, compared to other CNN architectures, is its ability to adapt its size in order to control the complexity of the system to be utilized in the study. In the MobileNetV1 model, the top layer was replaced by two fully connected layers and a final sigmoid output layer. To avoid overfitting, they used a data augmentation pipeline to transform images randomly before each training step.

Additionally, the pipeline changes the brightness in a random manner. Specifying a dropout rate of 10% for all layers and stopping early after 20 epochs of validation is another addition to avoiding overfitting. In this context, Santos *et al.* [98] built an ensemble of five CNNs (VGG-16, VGG-19, Resnet-50, InceptionV3, and Densenet-201). Using batch normalization and dropout operations, they fine-tuned CNNs and evaluated different configurations of fully connected layers on 8250 images with varied resolution, contrast, etc. of other characteristics. The individual CNNs worked well and furthermore there was a significant increase in success rates when the five CNNs were combined. Yogapriya *et al.* [99] developed a diabetic foot infection network (DFINET) to systematically assess diabetic foot ulcer images for the presence and absence of infection. This network is composed of 22 layers, including a fully connected layer with a dropout connection, a normalization layer, and a parallel convolution layer.

Moreover, Das *et al.* [100] proposed a new DCNN that is based on ResKNet and consists of unique residual blocks of 2D convolution, batch normalization, and leaky reLU with skip connections with achievement greater than 95% in every evaluation metric. Later, Das*et al.* [101] introduced a CNN architecture with a deep residual block that extracts a wide range of high-level features that are combined with different machine learning algorithms. A value of 96.50% is achieved by the logistic regression algorithm.

Reyes *et al.* [102] proposed an automated classification method for abnormal skin (DFU) versus normal skin (healthy skin) that uses a Novel Deep Convolutional Neural Network (CNN) architecture named DFU_VIRNet. The proposed CNN is trained and validated using visible and infrared (thermography) images. They also present a new method that relies on estimation maps in order to detect high-risk zones in patients who are likely to develop DFU. Nagaraju *et al.* [103] developed a novel Sparrow Search Optimization (SSO) technique using deep learning to detect and classify diabetic foot ulcers (SSODL-DFUDC). To accomplish this, the proposed technique employs the Inception-ResNet-v2 model for generating feature vectors. Using the SSO algorithm for the optimal hyperparameter selection of the Inception-ResNet-v2 model can be a simple and erroneous alternative to trial and error manual hyperparameter tuning. As a result, the overall DFU classification results will be improved. Biswas *et al.* [104] introduced a new framework for classifying images of healthy and ulcerated skin using publicly available repositories called "DFU_MultiNet.". Three well-known pre-trained CNN models—VGG19, DenseNet201, and NasNetMobile are used in the suggested technique to extract features from foot data. To build an effective hybrid network, these extracted data are combined via a summation layer. For the accurate classification of diabetic foot ulcer (DFU) images, a novel approach utilizing deep neural networks and machine learning is presented by Toofanee *et al.* [105]. Within a Siamese Neural Network (SNN) Architecture, a CNN and Vision Image Transformer (ViT) are harnessed for the proposed method. The model correctly categorizes DFU images into

four distinct classes based on similarity learning: None, Infection, Ischemia, or Both. Furthermore, Das *et al.* [106] introduced a novel Hybrid Convolutional Neural Network (HCNNet), which integrates multiple hybridised blocks including inception, residuals, dense layers, and squeeze-and-excite (SE) blocks. For optimum performance, the proposed HCNNet is trained several times using a variety of optimizer and learning rate settings. Fadhel *et al.* [107] presented a method for real-time classification of diabetic foot ulcers using deep learning techniques combined with parallel hardware computational tools. By leveraging advanced neural networks and efficient hardware, the approach significantly improves the speed and accuracy of ulcer detection, offering potential for enhanced clinical diagnostics and patient care.

Recently, Patel *et al.* [108] presented a multi-modal deep learning framework that combined wound images and location data to improve categorization accuracy. Using several test sets such as AZH and Medetec, it achieved 74.79–100% correct rates. Almufadi *et al.* [109] proposed E-DFu-Net, a convolutional network that scored 97% on ischemia classification and 92% for infection. It stands as a noteworthy advancement in DFU detection using transfer learning methods. Ajay *et al.* [110] developed Dense-ShuffleGCANet, which incorporates mechanisms driven by attention into the algorithms for robust classification. In addition, it has spatio-dimensional features of these patients' cases available. Karthik *et al.* [111] described a Swin Transformer model with multi-scale attention that obtained 80% F1 score on DFUC-2021, yet stressed its readability through Grad-CAM explainability. Ullah *et al.* [112] proposed Eff-ReLU-Net, replacing Swish activation with ReLU and incorporating dense layers. It achieved 92.33% accuracy on Medetec, and about 90% on AZH datasets. It emphasizes efficiency and generalization. Reis *et al.* [113] studied CNN fairness across different skin tones in the task of classifying DFU, displaying disparities while simultaneously calling for AI systems that are inclusive. Maurya *et al.* [114] proposed MCTFWC, a hybrid CNN-transformer system achieving excellent performance and robustness in wound classification. Fitriah *et al.* [115] used a modified version of MobileNetV2 for DFU severity classification in people with Type 2 diabetes, showing the method's efficiency in low-resource settings. Bansal *et al.* [116] employed ML classifiers with multivariate feature extraction for the analysis of DFU, achieving encouraging accuracy but noting ML limitations compared to deep learning. Karthik *et al.* [117] finally discussed the notable accuracy and robustness of Dense-ShuffleGCANet, incorporating attention mechanisms and spatio-dimensional features for advanced DFU classification. These reports together show that novel deep learning architectures and advances in fairness, explainability and robustness are being introduced across multiple threads of work in this area.

1.3.2. Thermal/Thermographic Image-based Approaches

Thermography is an non-invasive imaging method utilized to view thermal traces using an infrared (IR) camera. Temperature variations in vascular tissues can be qualitatively and visually captured by this procedure. In Adam *et al.* [118], a thermogram of the plantar foot is taken from 33 healthy people and 33 individuals with type 2 diabetes. These photographs are analyzed using discrete wavelet transform (DWT) and higher order spectra (HOS) techniques. Analyzed images are used to extract texture and entropy features. Using SVM classification, the DWT and HOS features were ranked using t-values before performing the classification.

As the occurrence of a significant increase in skin temperature is an early sign of the formation of a DFU, Vardasca *et al.* [119] processed and classified infrared thermal (IRT) images of 56 DFU patients at early stages, and an accuracy of 92.5% was achieved by using k-NN.

Statistical analysis revealed that thermal asymmetry values and thermal recovery values did not differ significantly in all Regions Of Interest (ROIs), with the exception of the one located at the medial forefoot [120]. Vardasca *et al.* [121] extended the above study by analyzing the dynamic IRT plantar foot examination images of 39 active DFU patients by measuring the mean temperature of ROI. They built a decision support system using ML algorithms (ANN, SVM, k-NN), to classify the data into neuro-ischemic and ischemic. Furthermore, Selle *et al.* [122] used SVM classifiers to calculate gray-level texture features from foot thermogram images in the form of a matrix based on normal and diabetic foot temperature values.

There has been no research conducted on the detection of peripheral arterial disease (PAD) from the top of the foot. Therefore, Padierna *et al.* [123] proposed an analysis of relevant features extracted from IRT images of the upper side of the foot and toes that can be used for this type of characterization. Using SVM, they developed an ML system for two groups of Mexican participants.

The distribution of changes in plantar temperature in diabetic patients does not follow a particular pattern, making it difficult to measure them. The first study of DL networks applied to the classification of diabetic foot thermograms was by Cruz *et al.* [124]. Currently, researchers are interested in improving the effectiveness of the analysis and classification methods for detecting abnormal changes in plantar temperature. They evaluated popular frameworks in the mode of transfer learning, including AlexNet and GoogleNet. Moreover, they introduced a new architecture for multi-level classification, which is self-trained and capable of achieving higher accuracy and quality measures. Additionally, Jain *et al.* [125] analyzed the performance of transfer learning-based DL networks (AlexNet [126] and Resnet-101 [127]) in foot thermogram classification into CG (Control Group) and DM (Diabetic Mellitus) groups. Moreover, they proposed a novel and enhanced technique (ProNet) by combining the best advantages of both AlexNet and ResNet [128]. The results of this work can be summarized as follows: AlexNet has greater accuracy (96.8%) than that obtained in the existing work. ResNet101 gives better results than AlexNet (accuracy of 97.9%). ProNet achieves improved performance in terms of accuracy (as high as 98.9%) and other metrics like precision, specificity, and F1 Score. Furthermore, they evaluated the performance of transfer learning DL networks (DarkNet-19 [129] and DarkNet-53 [130]) in the multiclass classification of DFUs based on Thermal Change Indices (TCIs). Lastly, an improved and novel methodology (Pro-Multi-Net), is proposed. The best features of the combined design include the features of both DarkNet-19 and DarkNet-53. They concluded that all three methods implemented have higher accuracy than the techniques applied in the literature. Total accuracy of 92.8% is also achieved by DarkNet-53 compared to DarkNet-19.

In Khandakar *et al.*2021 [131], ML-based scoring was compared to a state-of-the-art CNN on foot thermogram images, and a robust approach for identifying diabetic feet was presented. MobilenetV2, a comparatively shallow CNN model, obtained an F1 score of 95% for a thermogram image-based classification based on two feet, and AdaBoost Classifier obtained an F1 score of 97%. Moreover, Khandakar *et al.* [132] categorized a publicly available dataset by a temperature distribution parameter called thermal change index (TCI). Various machine-learning approaches were then used to classify the thermograms of the TCI-labeled dataset. To identify which algorithm is most effective for the classification of thermograms, a number of ML algorithms have been examined, including conventional algorithms and CNNs. In multiclass classification, the multilayer perceptron (MLP) classifier outperformed the literature-reported performance metrics with 90.1% accuracy.

Until that time, no ML-based techniques had been reported in the literature for analyzing thermograms to determine the severity of diabetic foot complications. In [133],

K-Means clustering is used to cluster diabetic foot ulcer severity risk based on available labeled diabetic thermograms. Using plantar foot temperature, the newly clustered dataset is verified by expert doctors for foot ulcer risk. ML networks are then tested on the newly labeled dataset for robustness. To classify thermograms based on severity, classical ML approaches combined with CNNs have been examined. With regards to severity stratification, the popular VGG 19 CNN model is found to have 95.08% accuracy, 95.08% precision, 95.09% sensitivity, 95.08% specificity, and 97.2% specificity, respectively.

Recently, two different classification models have been proposed and compared by Filipe *et al.* [134] for thermograms of diabetic and healthy feet. Both models use ML algorithms to detect and classify abnormal plantar temperature changes. The first model categorizes foot thermograms into four categories: healthy, diabetic, and other. In the second model, the foot is classified as having diabetes or normal in the first stage. In comparison to Model 1, Model 2 offers superior performance and is more accurate in identifying healthy individuals and diabetics.

Some other emerging research work focuses on Decision Fusion (DF). Munadi *et al.* [135] propose a novel framework for DFU classification based on thermal imaging where DF incorporates the classification result from a parallel classifier. A novel DF method was used to fuse both datasets using plantar thermograms to increase accuracy. The DFU thermal images were classified into binary classes of positive and negative cases with 100% accuracy. They found that the addition of DF improved accuracy by approximately 3.4%.

Anaya *et al.* [136] aimed to investigate three state-of-the-art DL architectures, experimenting with convolutional, residual, and attention (Transformer) approaches to categorize diabetic foot thermography images into DM subjects. There were three conditions under which the models were trained. By modifying the images through the change in amplitude in the Fourier Transform, a novel method is proposed for the characterization of ulcer risk using thermographies.

khosa *et al.* [137] presented a comprehensive and comparative analysis of different DL and ML approaches for the classification of DFU feet. This study uses multilevel of thermogram data from DFU and non-DFU feet, at the level of an image, a patch, and a combined image-patch.

1.4. Combining different image types for classification models

Reyes-Luévano *et al.* [138] describe the first research to combine visible and infrared (thermography) images into DFU_VIRNet. This network consists of two parallel branches of convolution blocks with kernels of heterogeneous size (5×5 and 3×3) and in increasing order $(32, 64, 128, 256, and 512)$. Each convolution block was constructed using a 1×1 stride with ReLU activation. They have implemented a frozen (untrained) and pre-trained Xception model to accelerate the learning process. To create a new input image for the network structure, a new substructure is created which is called (GAP-2D-DLSA-IMG) and consists of a Global Average Pooling 2D layer (GAP-2D) and a Dense Layer with Sigmoid Activation (DLSA). The proposed architecture, DFU_VIRNet, was found to have high consistency across all performance metrics, significantly improving on the results of the previous state-of-the-art. It also reduced overfitting and eased the network's receptive field. The DFU_VIRNet feature maps have very limited spatial (geometric) meaning for humans, so the proposed estimation maps are essential to understanding the features learned by the model, and to estimating and visualizing zones that are likely to develop DFU.

1.5. Hybrid models (segmentation and classification)

1.5.1. Colour Image-based Approaches

There remains a need for a user-friendly system combining dimensional measurements and tissue classification for accurate wound healing assessment. The first objective was achieved by Wannous *et al.* [139] using uncalibrated vision techniques to compute a 3-D wound measurement model. Following unsupervised segmentation, they computed colour and texture descriptors to classify tissues. By identifying wound outlines and measuring area and volume, the results demonstrated increased repeatability and robustness.

Mukherjee *et al.* [140] aimed to develop a classification of three classes (granulation, necrotic, and slough) for chronic wounds (CW) using ML and image processing. Initially, they transformed the images to HSI (hue, saturation, and intensity) colour space, and then the component of colour channels was selected to achieve a greater degree of contrast. Next, six different segmented areas were founded from images using fuzzy divergence-based thresholding by minimizing edge ambiguity. A Bayesian classification algorithm and an SVM were used to train and test different CW images. The results show that 3rd order polynomial kernel SVM provided the highest accuracy.

One of the early trials of DFU detection was by Babu *et al.* [141]. In this study, an optimization technique based on particle swarm optimization (PSO) was used to segment diabetic wounds and classify them into three types of tissues, namely, granulation, necrotic, and slough. Following the segmentation, the Gray Level Co-occurrence Matrix (GLCM) was used to extract the different textural features. Two different classifiers were used for the classification of tissues, Naive Bayes and Hoeffding tree. From the experimental results it was found that the classification accuracy using Naive Bayes was better than the Hoeffding tree.

The researchers in Godeiro *et al.* [142] explored algorithms for segmenting wounds and utilizing CNNs for classifying tissue as necrotic, granulation, or slough. A colour space reduction methodology was presented that significantly improved the reported accuracies, specificities, sensitivities, and Dice coefficients of four architectures: U-Net, Segnet, FCN8, and FCN32. Wijesinghe *et al.* [143] proposed a prototype system, called the Intelligent Diabetic Assistant (IDA), which is an autonomous system that determines the diagnosis and priority of treatment based on the observations displayed on the screen. This system consists of knowledge-based modules (pre-trained Deep ensemble CNN, and Mask R-CNN) for the classification of foot ulcer severity levels and clinical decision support, as well as near real-time detection of foot ulcer boundaries.

Zhou *et al.* [144] developed a diagnostic model for DFU segmentation and classification using deep learning on 671 annotated images. The model compared three other models including Mask2Former, Deeplabv3Plus, and Swin-Transformer. Mask2Former one achieved the best performance, attaining a mean IoU of 65.79% and a wound segmentation IoU of 85.88% with an accuracy of 91.85% and AUC of 0.9429 for Wagner grade classification. The study showed that Mask2Former identified the key ulcer components such as necrotic tissue since the former model did not identify the same and even identified the tendons and bones. Strong end-to-end robust preprocessing that included resizing and strong data augmentation to boost model performance would provide a viable automated way of DFU grading ready for clinical use.

1.5.2. Thermal/Thermographic Image-based Approaches

To analyze foot pressure, Sudarvizhi *et al.* [145] embedded load cell sensors inside a mat and used WEKA data mining software for classification and feature selection [146]. Segmentation was performed using a bi-model histogram with two different peaks. Maldonado *et al.* [147] proposed a method for the detection and classification of temperature

differences in the foot sole zone. Through the application of deep-learning techniques, a mask R-CNN model was retrained to segment visible-spectrum images, which were adjusted with 141 images to segment foot soles. These results were used over the temperature matrix to isolate foot surface temperatures. This method employs a step-by-step algorithm to compare homologous sole regions, and a convolutional function with a two-dimensional Gaussian function to classify them.

2. The Datasets Used in Literature

Generally, AI systems are learning from one or more Datasets (DS) varying in size from small to large ones. These DSs have been formed in collaboration with doctors/medical specialists to provide high-quality, well-selected images, previously analyzed, labeled, and potentially segmented by the specialists from the DFU domain. Our study aims to describe the growth trends of AI systems that are able to diagnose, segment, or detect certain diabetic foot symptoms based on existing papers in the literature. The results described in these AI papers are possible because of the availability of public DSs. In this section, we will present some of most popular DSs which have been used in the most cited or recent papers from the DFU domain.

2.1. Different DFU Images

The focus of some of the earliest DFU research, in 2015, was based on computer vision methods (based on image processing algorithms). Conventional ML methods were used to analyse those images (Yap *et al.* [148,149]). They were the first researchers focused on the development of a new mobile app using "FootSnap," to standardize photographs of diabetic feet and to test its reliability on different occasions and between different operators. Then, Wang *et al.* [2] introduced the "Swift Wound" app which provides highly reliable and accurate wound measurements.

With the advent of DL in computer vision tasks, researchers began investigating the use of DL for DFU segmentation in 2016, and the first fully automated segmentation paper was published in 2017 by [3]. Yap *et al.* [150] presented challenges related to DFUs in a comprehensive evaluation of the state-of-the-art popular object detection frameworks used in DFU detection and included an overview of publicly available datasets. They provided an ensemble method and cascade attention. detNet was proposed for detecting DFU, and a comprehensive evaluation of deep learning algorithms on the DFUC2020 dataset was conducted. The Diabetic Foot Ulcers Grand Challenge (DFUC2020) dataset consists of 2000 training images, 200 validation images and 2000 testing images [6,83].

In 2021, another dataset was described for the Diabetic Foot Ulcers Grand Challenge (DFUC2021) Cassidy *et al.* [151]. To facilitate the development of semi-supervised and weakly-supervised DL techniques, DFUC2021 provides a greater number of diabetic foot ulcer patches, of which 5,955 are used for training, 5,734 are used for testing, and 3,994 are unlabeled. A 640×480 pixel dimension was used to reduce computational costs and improve the performance of DL methods. Recently, Yap *et al.* [152] provided a detailed review of the development of diabetic foot ulcer datasets over the past decade. Boualla *et al.* [153] introduced a STANDUP research database that contains 415 multispectral plantar foot images (thermal and RGB images) from healthy (125 images) and diabetic subjects (290 images) (the healthy group was collected from France and Morocco and the diabetic patient group was from Peru). In Table 1, We show the details of DFU datasets and their characteristics and usage.

Table 1. Main characteristics and usage of DFU images datasets in literature [152] including author, year, dataset employed, resolution of images used, type of targeted task, and distribution of data.

Author[ref.]	Year	Dataset Name	Resolution (pixels)	Task	Train	Test	Total
Thomas [154]	2014	Medetec	560 x 391<br>224 x 224	segmentation	152	8	160
Goyal *et al.* [4]	2018	DFU-Part (A)(I)	varied	classification	NA	NA	1,679
Hernandez-Contreras *et al.* [155]	2019	PLANTAR THERMO-GRAM	320 × 240	segmentation	NA	NA	1670
Alzubaidi *et al.* [85]	2020	DFU	224 × 224	classification	NA	NA	493
Goyal *et al.* [6]	2020	DFU-Part (B)(II)	256 × 256	classification	NA	NA	1,459
Cassidy *et al.* [83]	2020	DFUC2020	640 × 480	detection	2,000	2,000	4,000
Wang *et al.* [36]	2020	AZH Chronic wound dataset	224 × 224	segmentation	831	278	1,109
Lucas *et al.* [156]	2020	ESCALE	NA	segmentation	NA	NA	92
Krkecichwost *et al.* [157]	2021	WoundDB	Thermal<br>320 ×240<br>RGB<br>1920 ×1080	seg<br>*backslash*<br>class	NA	NA	188
Wang *et al.* [29]	2021	FUSeg Challenge	512 × 512	segmentation	1,010	200	1,210
Yap *et al.* [90]	2021	DFUC2021	224 × 224	classification	5,955	5,734	$15,683^{*}$
Bouallal *et al.* [153]	2022	STANDUP	Thermal<br>160×120<br>RGB<br>1440×1080	segmentation	NA	NA	415
Kendrick *et al.* [55]	2022	DFUC2022	640 × 480	segmentation	2000	2000	4000

* 3.994 patches are unlabelled; NA indicates unknown

References

1. Wang, L.; Pedersen, P.C.; Agu, E.; Strong, D.M.; Tulu, B. Area determination of diabetic foot ulcer images using a cascaded two-stage SVM-based classification. *IEEE Transactions on Biomedical Engineering* **2016**, *64*, 2098–2109.
2. Wang, S.C.; Anderson, J.A.; Evans, R.; Woo, K.; Beland, B.; Sasseville, D.; Moreau, L. Point-of-care wound visioning technology: Reproducibility and accuracy of a wound measurement app. *PloS one* **2017**, *12*, e0183139.
3. Goyal, M.; Yap, M.H.; Reeves, N.D.; Rajbhandari, S.; Spragg, J. Fully convolutional networks for diabetic foot ulcer segmentation. In Proceedings of the 2017 IEEE International Conference on Systems, Man, and Cybernetics (SMC), 2017, pp. 618–623.
4. Goyal, M.; Reeves, N.D.; Davison, A.K.; Rajbhandari, S.; Spragg, J.; Yap, M.H. Dfunet: Convolutional neural networks for diabetic foot ulcer classification. *IEEE Transactions on Emerging Topics in Computational Intelligence* **2018**, *4*, 728–739.
5. Goyal, M.; Reeves, N.D.; Rajbhandari, S.; Yap, M.H. Robust methods for real-time diabetic foot ulcer detection and localization on mobile devices. *IEEE journal of biomedical and health informatics* **2018**, *23*, 1730–1741.
6. Goyal, M.; Reeves, N.D.; Rajbhandari, S.; Ahmad, N.; Wang, C.; Yap, M.H. Recognition of ischaemia and infection in diabetic foot ulcers: Dataset and techniques. *Computers in Biology and Medicine* **2020**, *117*, 103616.
7. Han, A.; Zhang, Y.; Li, A.; Li, C.; Zhao, F.; Dong, Q.; Liu, Q.; Liu, Y.; Shen, X.; Yan, S.; et al. Efficient refinements on YOLOv3 for real-time detection and assessment of diabetic foot Wagner grades. *arXiv preprint arXiv:2006.02322* **2020**.
8. Brüngel, R.; Friedrich, C.M. DETR and YOLOv5: exploring performance and self-training for diabetic foot ulcer detection. In Proceedings of the 2021 IEEE 34th International Symposium on Computer-Based Medical Systems (CBMS). IEEE, 2021, pp. 148–153.
9. Ge, Z.; Liu, S.; Wang, F.; Li, Z.; Sun, J. Yolox: Exceeding yolo series in 2021. *arXiv preprint arXiv:2107.08430* **2021**.
10. Jocher, G.; Nishimura, K.; Mineeva, T.; Vilariño, R. yolov5. *Code repository* **2020**.
11. Cassidy, B.; Reeves, N.D.; Pappachan, J.M.; Ahmad, N.; Haycocks, S.; Gillespie, D.; Yap, M.H. A cloud-based deep learning framework for remote detection of diabetic foot ulcers. *IEEE Pervasive Computing* **2022**.
12. Thotad, P.N.; Bharamagoudar, G.R.; Anami, B.S. Diabetic foot ulcer detection using deep learning approaches. *Sensors International* **2023**, *4*, 100210.
13. Sarmun, R.; Chowdhury, M.E.; Murugappan, M.; Aqel, A.; Ezzuddin, M.; Rahman, S.M.; Khandakar, A.; Akter, S.; Alfkey, R.; Hasan, A. Diabetic foot ulcer detection: combining deep learning models for improved localization. *Cognitive computation* **2024**, *16*, 1413–1431. https://doi.org/https://doi.org/10.1007/s12559-024-10267-3.
14. Sendilraj, V.; Pilcher, W.; Choi, D.; Bhasin, A.; Bhadada, A.; Bhadadaa, S.K.; Bhasin, M. DFUCare: Deep learning platform for diabetic foot ulcer detection, analysis, and monitoring. *Frontiers in Endocrinology* **2024**, *15*, 1386613. https://doi.org/https://doi.org/10.3389/fendo.2024.1386613.
15. Biswas, S.; Mostafiz, R.; Uddin, M.S.; Paul, B.K. XAI-FusionNet: Diabetic foot ulcer detection based on multi-scale feature fusion with explainable artificial intelligence. *Heliyon* **2024**, *10*. https://doi.org/https://doi.org/10.1016/j.heliyon.2024.e31228.
16. El-Kady, A.M.; Abbassy, M.M.; Ali, H.H.; Ali, M.F. Advancing diabetic foot ulcer detection based on resnet and gan integration. *Journal of Theoretical and Applied Information Technology* **2024**, *102*, 2258–2268. https://doi.org/https://www.jatit.org/volumes/Vol102No6/2Vol102No6.pdf.
17. Azeem, M.; Zaman, M.; Akhunzada, A.; Kehkashan, T.; Ashraf, I.; Rehman, A. Optimizing Diabetic Foot Ulcer Detection: Leveraging SSD and YOLO Architectures. In Proceedings of the 2024 IEEE 21st International Conference on Smart Communities: Improving Quality of Life using AI, Robotics and IoT (HONET), 2024, pp. 195–200. https://doi.org/https://doi.org/10.1109/HONET63146.2024.10822990.
18. Verma, G. Leveraging smart image processing techniques for early detection of foot ulcers using a deep learning network. *Polish Journal of Radiology* **2024**, *89*, e368. https://doi.org/https://doi.org/10.5114/pjr/189412.

19. Busaranuvong, P.; Agu, E.; Kumar, D.; Gautam, S.; Fard, R.S.; Tulu, B.; Strong, D. Guided Conditional Diffusion Classifier (ConDiff) for Enhanced Prediction of Infection in Diabetic Foot Ulcers. *IEEE Open Journal of Engineering in Medicine and Biology* **2024**, *6*, 20–27. https://doi.org/https://doi.org/10.1109/OJEMB.2024.3453060.
20. Eldin, A.S.; Ahmoud, A.S.; Hamza, H.M.; Ardah, H. Enhancing Early Detection of Diabetic Foot Ulcers Using Deep Neural Networks. *Diagnostics* **2025**, *15*, 1996. https://doi.org/https://doi.org/10.3390/diagnostics15161996.
21. Rathore, P.S.; Kumar, A.; Nandal, A.; Dhaka, A.; Sharma, A.K. A feature explainability-based deep learning technique for diabetic foot ulcer identification. *Scientific Reports* **2025**, *15*, 6758. https://doi.org/https://doi.org/10.3390/diagnostics15161996.
22. Debnath, S.; Khurana, A.; Senbagavalli, M.; Naik, S.; Chandra Patni, J.; Mishra, P.K.; Kishore, J. Sustainable AI for diabetic foot ulcer detection: a deep learning approach for early diagnosis. *Discover Applied Sciences* **2025**, *7*, 1012. https://doi.org/https://doi.org/10.1007/s42452-025-07601-1.
23. Mahmud, M.I.; Reza, M.S.; Akash, M.O.A.; Elias, F.; Ahmed, N. DFU_DIALNet: Towards reliable and trustworthy diabetic foot ulcer detection with synergistic confluence of Grad-CAM and LIME. *Plos one* **2025**, *20*, e0330669. https://doi.org/https://doi.org/10.1371/journal.pone.0330669.
24. Girmaw, D.W.; Taye, G.B. MobileNetV2 model for detecting and grading diabetic foot ulcer. *Discover Applied Sciences* **2025**, *7*, 1–19. https://doi.org/https://doi.org/10.1007/s42452-025-06745-4.
25. Pradhana, W.M.A.A.; Pradipta, G.A.; Huizen, R.R. Combination of CNN and SMOTE-IPF for Early Detection of Diabetes Patients in Thermogram Images. *Jurnal Nasional Pendidikan Teknik Informatika: JANAPATI* **2025**, *14*. https://doi.org/https://doi.org/10.23887/janapati.v14i2.83145.
26. Nag, U.; Upadhayay, M.; Gupta, T. Detecting Diabetic Foot Complications using Infrared Thermography and Machine Learning. In Proceedings of the International Conference on Graphics and Signal Processing, 2021, pp. 41–46.
27. Dremin, V.; Marcinkevics, Z.; Zherebtsov, E.; Popov, A.; Grabovskis, A.; Kronberga, H.; Geldnere, K.; Doronin, A.; Meglinski, I.; Bykov, A. Skin complications of diabetes mellitus revealed by polarized hyperspectral imaging and machine learning. *IEEE Transactions on Medical Imaging* **2021**, *40*, 1207–1216.
28. Lindberg, J.W. Predicting clinical outcomes in a diabetic foot ulcer population using fluorescence imaging. *Advances in Skin & Wound Care* **2021**, *34*, 596–601.
29. Wang, C.; Mahbod, A.; Ellinger, I.; Galdran, A.; Gopalakrishnan, S.; Niezgoda, J.; Yu, Z. FUSeg: The Foot Ulcer Segmentation Challenge. *arXiv preprint arXiv:2201.00414* **2022**.
30. Cui, C.; Thurnhofer-Hemsi, K.; Soroushmehr, R.; Mishra, A.; Gryak, J.; Domínguez, E.; Najarian, K.; López-Rubio, E. Diabetic wound segmentation using convolutional neural networks. In Proceedings of the Annual International Conference of the IEEE Engineering in Medicine and Biology Society (EMBC), 2019, pp. 1002–1005.
31. Ohura, N.; Mitsuno, R.; Sakisaka, M.; Terabe, Y.; Morishige, Y.; Uchiyama, A.; Okoshi, T.; Shinji, I.; Takushima, A. Convolutional neural networks for wound detection: the role of artificial intelligence in wound care. *Journal of Wound Care* **2019**, *28*, S13–S24.
32. Ronneberger, O.; Fischer, P.; Brox, T. U-net: Convolutional networks for biomedical image segmentation. In Proceedings of the International Conference on Medical image computing and computer-assisted intervention. Springer, 2015, pp. 234–241.
33. Simonyan, K.; Zisserman, A. Very deep convolutional networks for large-scale image recognition. *arXiv preprint arXiv:1409.1556* **2014**.
34. Gamage, H.; Wijesinghe, W.; Perera, I. Instance-based segmentation for boundary detection of neuropathic ulcers through Mask-RCNN. In Proceedings of the International Conference on Artificial Neural Networks. Springer, 2019, pp. 511–522.
35. He, K.; Gkioxari, G.; Dollár, P.; Girshick, R. Mask r-cnn. In Proceedings of the Proceedings of the IEEE international conference on computer vision, 2017, pp. 2961–2969.

36. Wang, C.; Anisuzzaman, D.; Williamson, V.; Dhar, M.K.; Rostami, B.; Niezgoda, J.; Gopalakrishnan, S.; Yu, Z. Fully automatic wound segmentation with deep convolutional neural networks. *Scientific Reports* **2020**, *10*, 1–9.
37. Sandler, M.; Howard, A.; Zhu, M.; Zhmoginov, A.; Chen, L.C. Mobilenetv2: Inverted residuals and linear bottlenecks. In Proceedings of the Proceedings of the IEEE conference on computer vision and pattern recognition, 2018, pp. 4510–4520.
38. Jawahar, M.; Anbarasi, L.J.; Jasmine, S.G.; Narendra, M. Diabetic foot ulcer segmentation using color space models. In Proceedings of the 2020 5th international conference on communication and electronics systems (ICCES). IEEE, 2020, pp. 742–747.
39. Rania, N.; Douzi, H.; Yves, L.; Sylvie, T. Semantic segmentation of diabetic foot ulcer images: dealing with small dataset in DL approaches. In Proceedings of the International Conference on Image and Signal Processing. Springer, 2020, pp. 162–169.
40. Muñoz, P.; Rodríguez, R.; Montalvo, N. Automatic segmentation of diabetic foot ulcer from mask region-based convolutional neural networks. *Journal of Biomedical Research and Clinical Investigation* **2020**, *1*.
41. Mahbod, A.; Ecker, R.; Ellinger, I. Automatic Foot Ulcer segmentation Using an Ensemble of Convolutional Neural Networks. *arXiv preprint arXiv:2109.01408* **2021**.
42. Huang, C.H.; Wu, H.Y.; Lin, Y.L. Hardnet-mseg: A simple encoder-decoder polyp segmentation neural network that achieves over 0.9 mean dice and 86 fps. *arXiv preprint arXiv:2101.07172* **2021**.
43. Galdran, A.; Carneiro, G.; Ballester, M.A.G. Double encoder-decoder networks for gastrointestinal polyp segmentation. In Proceedings of the International Conference on Pattern Recognition. Springer, 2021, pp. 293–307.
44. Chaurasia, A.; Culurciello, E. Linknet: Exploiting encoder representations for efficient semantic segmentation. In Proceedings of the 2017 IEEE Visual Communications and Image Processing (VCIP). IEEE, 2017, pp. 1–4.
45. Tan, M.; Le, Q. Efficientnet: Rethinking model scaling for convolutional neural networks. In Proceedings of the International conference on machine learning. PMLR, 2019, pp. 6105–6114.
46. Hernández, A.; Arteaga-Marrero, N.; Villa, E.; Fabelo, H.; Callicó, G.M.; Ruiz-Alzola, J. Automatic Segmentation Based on Deep Learning Techniques for Diabetic Foot Monitoring Through Multimodal Images. In Proceedings of the International Conference on Image Analysis and Processing, 2019, pp. 414–424.
47. Wu, Z.; Su, L.; Huang, Q. Cascaded partial decoder for fast and accurate salient object detection. In Proceedings of the Proceedings of the IEEE/CVF conference on computer vision and pattern recognition, 2019, pp. 3907–3916.
48. Chang, C.W.; Christian, M.; Chang, D.H.; Lai, F.; Liu, T.J.; Chen, Y.S.; Chen, W.J. Deep learning approach based on superpixel segmentation assisted labeling for automatic pressure ulcer diagnosis. *PloS one* **2022**, *17*, e0264139.
49. Chen, L.C.; Zhu, Y.; Papandreou, G.; Schroff, F.; Adam, H. Encoder-decoder with atrous separable convolution for semantic image segmentation. In Proceedings of the Proceedings of the European conference on computer vision (ECCV), 2018, pp. 801–818.
50. LinTY, D.; GirshickR, H.; HariharanB, B. Featurepyramidnet worksforobjectdetection. *ProceedingsoftheIEEECon ference on Computer Vision and Pattern Recognition* **2017**, *2117*, 2125.
51. Kirillov, A.; Girshick, R.; He, K.; Dollár, P. Panoptic feature pyramid networks. In Proceedings of the Proceedings of the IEEE/CVF conference on computer vision and pattern recognition, 2019, pp. 6399–6408.
52. Chitra, T.; Sundar, C.; GOPALAKRISHNAN, S. Investigation and classification of chronic wound tissue images using random forest algorithm (RF). *International Journal of Nonlinear Analysis and Applications* **2022**, *13*, 643–651.
53. Biau, G.; Scornet, E. A random forest guided tour. *Test* **2016**, *25*, 197–227.
54. Liao, T.Y.; Yang, C.H.; Lo, Y.W.; Lai, K.Y.; Shen, P.H.; Lin, Y.L. HarDNet-DFUS: An Enhanced Harmonically-Connected Network for Diabetic Foot Ulcer Image Segmentation and Colonoscopy Polyp Segmentation. *arXiv preprint arXiv:2209.07313* **2022**.

55. Kendrick, C.; Cassidy, B.; Pappachan, J.M.; O'Shea, C.; Fernandez, C.J.; Chacko, E.; Jacob, K.; Reeves, N.D.; Yap, M.H. Translating Clinical Delineation of Diabetic Foot Ulcers into Machine Interpretable Segmentation. *arXiv preprint arXiv:2204.11618* **2022**.
56. Huang, H.N.; Zhang, T.; Yang, C.T.; Sheen, Y.J.; Chen, H.M.; Chen, C.J.; Tseng, M.W. Image segmentation using transfer learning and Fast R-CNN for diabetic foot wound treatments. *Frontiers in Public Health* **2022**, *10*.
57. Heras-Tang, A.; Valdes-Santiago, D.; León-Mecías, Á.M.; Díaz-Romañach, M.L.B.; Mesejo-Chiong, J.A.; Cabal-Mirabal, C. Diabetic foot ulcer segmentation using logistic regression, DBSCAN clustering and mathematical morphology operators. *Electronic Letters on Computer Vision and Image Analysis* **2022**, *21*, 22–39.
58. Lan, T.; Li, Z.; Chen, J. FusionSegNet: Fusing global foot features and local wound features to diagnose diabetic foot. *Computers in Biology and Medicine* **2023**, *152*, 106456.
59. Jishnu, P.; BK, S.K.; Jayaraman, S. Automatic foot ulcer segmentation using conditional generative adversarial network (AFSegGAN): A wound management system. *PLOS Digital Health* **2023**, *2*, e0000344.
60. Jiao, C.; Zhao, X.; Li, L.; Wang, C.; Chen, Y. UFOS-Net leverages small-scale feature fusion for diabetic foot ulcer segmentation. *Scientific Reports* **2025**, *15*, 29317. https://doi.org/https://doi.org/10.1038/s41598-025-12442-4.
61. Niri, R.; Zahia, S.; Stefanelli, A.; Sharma, K.; Probst, S.; Pichon, S.; Chanel, G. Wound segmentation with U-Net using a dual attention mechanism and transfer learning. *Journal of imaging informatics in medicine* **2025**, pp. 1–15. https://doi.org/https://doi.org/10.1007/s10278-025-01386-w.
62. Kaabouch, N.; Hu, W.C.; Chen, Y.; Anderson, J.W.; Ames, F.; Paulson, R. Predicting neuropathic ulceration: analysis of static temperature distributions in thermal images. *Journal of Biomedical Optics* **2010**, *15*, 061715.
63. Bouallal, D.; Bougrine, A.; Douzi, H.; Harba, R.; Canals, R.; Vilcahuaman, L.; Arbanil, H. Segmentation of plantar foot thermal images: Application to diabetic foot diagnosis. In Proceedings of the International Conference on Systems, Signals and Image Processing (IWSSIP). IEEE, 2020, pp. 116–121.
64. Bouallal, D.; Douzi, H.; Harba, R. Diabetic foot thermal image segmentation using Double Encoder-ResUnet (DE-ResUnet). *Journal of Medical Engineering & Technology* **2022**, pp. 1–15.
65. Bougrine, A.; Harba, R.; Canals, R.; Ledee, R.; Jabloun, M. On the segmentation of plantar foot thermal images with Deep Learning. In Proceedings of the European Signal Processing Conference (EUSIPCO). IEEE, 2019, pp. 1–5.
66. Bougrine, A.; Harba, R.; Canals, R.; Ledee, R.; Jabloun, M.; Villeneuve, A. Segmentation of Plantar Foot Thermal Images Using Prior Information. *Sensors* **2022**, *22*, 3835.
67. Long, J.; Shelhamer, E.; Darrell, T. Fully convolutional networks for semantic segmentation. In Proceedings of the Proceedings of the IEEE conference on computer vision and pattern recognition, 2015, pp. 3431–3440.
68. Badrinarayanan, V.; Kendall, A.; Cipolla, R. Segnet: A deep convolutional encoder-decoder architecture for image segmentation. *IEEE transactions on pattern analysis and machine intelligence* **2017**, *39*, 2481–2495.
69. Alshayeji, M.H.; Sindhu, S.C.; et al. Early detection of diabetic foot ulcers from thermal images using the bag of features technique. *Biomedical Signal Processing and Control* **2023**, *79*, 104143.
70. Keerthi, S.S.; Shevade, S.K.; Bhattacharyya, C.; Murthy, K.R.K. Improvements to Platt's SMO algorithm for SVM classifier design. *Neural computation* **2001**, *13*, 637–649.
71. Abdullah, A.A.; Jing, T.W.; Sie, C.A.; Yusuf, N.; Zakaria, A.; Omar, M.I.; Shakaff, A.Y.M.; Adom, A.H.; Kamarudin, L.M.; Juan, Y.E.; et al. Rapid identification method of aerobic bacteria in diabetic foot ulcers using electronic nose. *Advanced Science Letters* **2014**, *20*, 37–41.
72. Yusuf, N.; Zakaria, A.; Omar, M.I.; Shakaff, A.Y.M.; Masnan, M.J.; Kamarudin, L.M.; Abdul Rahim, N.; Zakaria, N.Z.I.; Abdullah, A.A.; Othman, A.; et al. In-vitro diagnosis of single and poly microbial species targeted for diabetic foot infection using e-nose technology. *BMC Bioinformatics* **2015**, *16*, 1–12.
73. Specht, D.F. Probabilistic neural networks. *Neural networks* **1990**, *3*, 109–118.

74. Jung, K.; Covington, S.; Sen, C.K.; Januszyk, M.; Kirsner, R.S.; Gurtner, G.C.; Shah, N.H. Rapid identification of slow healing wounds. *Wound Repair and Regeneration* **2016**, *24*, 181–188.
75. Botros, F.S.; Taher, M.F.; ElSayed, N.M.; Fahmy, A.S. Prediction of diabetic foot ulceration using spatial and temporal dynamic plantar pressure. In Proceedings of the Cairo international biomedical engineering conference (CIBEC), 2016, pp. 43–47.
76. Gamage, C.; Wijesinghe, I.; Perera, I. Automatic scoring of diabetic foot ulcers through deep CNN based feature extraction with low rank matrix factorization. In Proceedings of the International Conference on Bioinformatics and Bioengineering (BIBE), 2019, pp. 352–356.
77. Patel, S.; Patel, R.; Desai, D. Diabetic foot ulcer wound tissue detection and classification. In Proceedings of the 2017 international conference on innovations in information, embedded and communication systems (ICIIECS). IEEE, 2017, pp. 1–5.
78. Kasbekar, P.U.; Goel, P.; Jadhav, S.P. A decision tree analysis of diabetic foot amputation risk in Indian patients. *Frontiers in Endocrinology* **2017**, *8*, 25.
79. Liu, Q.; Zhao, J. The Classification of Diabetic Foot Based on Faster. In Proceedings of the International Conference on Computer Vision, Image and Deep Learning (CVIDL). IEEE, 2020, pp. 585–591.
80. Ren, S.; He, K.; Girshick, R.; Sun, J. Faster r-cnn: Towards real-time object detection with region proposal networks. *Advances in neural information processing systems* **2015**, *28*.
81. Guo, J.; Gould, S. Deep CNN ensemble with data augmentation for object detection. *arXiv preprint arXiv:1506.07224* **2015**.
82. Amin, J.; Sharif, M.; Anjum, M.A.; Khan, H.U.; Malik, M.S.A.; Kadry, S. An integrated design for classification and localization of diabetic foot ulcer based on CNN and YOLOv2-DFU models. *IEEE Access* **2020**, *8*, 228586–228597.
83. Cassidy, B.; Reeves, N.D.; Pappachan, J.M.; Gillespie, D.; O'Shea, C.; Rajbhandari, S.; Maiya, A.G.; Frank, E.; Boulton, A.J.; Armstrong, D.G.; et al. The DFUC 2020 dataset: Analysis towards diabetic foot ulcer detection. *touchREVIEWS in Endocrinology* **2021**, *17*, 5.
84. Tan, M.; Pang, R.; Le, Q.V. Efficientdet: Scalable and efficient object detection. In Proceedings of the Proceedings of the IEEE/CVF conference on computer vision and pattern recognition, 2020, pp. 10781–10790.
85. Alzubaidi, L.; Fadhel, M.A.; Oleiwi, S.R.; Al-Shamma, O.; Zhang, J. DFU_QUTNet: diabetic foot ulcer classification using novel deep convolutional neural network. *Multimedia Tools and Applications* **2020**, *79*, 15655–15677.
86. da Costa Oliveira, A.L.; de Carvalho, A.B.; Dantas, D.O. Faster R-CNN Approach for Diabetic Foot Ulcer Detection. In Proceedings of the International Joint Conference on Computer Vision, Imaging and Computer Graphics Theory and Applications (VISIGRAPP), 2021, pp. 677–684.
87. Galdran, A.; Carneiro, G.; Ballester, M.A.G. Convolutional nets versus vision transformers for diabetic foot ulcer classification. In Proceedings of the Diabetic Foot Ulcers Grand Challenge. Springer, 2021, pp. 21–29.
88. Xu, Y.; Han, K.; Zhou, Y.; Wu, J.; Xie, X.; Xiang, W. Classification of Diabetic Foot Ulcers Using Class Knowledge Banks. *Frontiers in Bioengineering and Biotechnology* **2021**, *9*.
89. Das, S.K.; Roy, P.; Mishra, A.K. DFU_SPNet: A stacked parallel convolution layers based CNN to improve Diabetic Foot Ulcer classification. *ICT Express* **2022**, *8*, 271–275.
90. Yap, M.H.; Cassidy, B.; Pappachan, J.M.; O'Shea, C.; Gillespie, D.; Reeves, N.D. Analysis towards classification of infection and ischaemia of diabetic foot ulcers. In Proceedings of the International Conference on Biomedical and Health Informatics (BHI), 2021, pp. 1–4.
91. Yap, M.H.; Hachiuma, R.; Alavi, A.; Brüngel, R.; Cassidy, B.; Goyal, M.; Zhu, H.; Rückert, J.; Olshansky, M.; Huang, X.; et al. Deep learning in diabetic foot ulcers detection: a comprehensive evaluation. *Computers in Biology and Medicine* **2021**, *135*, 104596.
92. Bloch, L.; Brüngel, R.; Friedrich, C.M. Boosting EfficientNets Ensemble Performance via Pseudo-Labels and Synthetic Images by pix2pixHD for Infection and Ischaemia Classification in Diabetic Foot Ulcers. In Proceedings of the Diabetic Foot Ulcers Grand Challenge. Springer, 2021, pp. 30–49.
93. Niri, R.; Douzi, H.; Lucas, Y.; Treuillet, S. A superpixel-wise fully convolutional neural network approach for diabetic foot ulcer tissue classification. In Proceedings of the International Conference on Pattern Recognition. Springer, 2021, pp. 308–320.

94. López-Cabrera, J.D.; Ruiz-Gonzalez, Y.; Díaz-Amador, R.; Taboada-Crispi, A. Automatic Classification of Diabetic Foot Ulcers Using Computer Vision Techniques. In Proceedings of the International Workshop on Artificial Intelligence and Pattern Recognition. Springer, 2021, pp. 290–299.
95. Al-Garaawi, N.; Ebsim, R.; Alharan, A.F.; Yap, M.H. Diabetic foot ulcer classification using mapped binary patterns and convolutional neural networks. *Computers in Biology and Medicine* **2022**, *140*, 105055.
96. Al-Garaawi, N.; Harbi, Z.; Morris, T. Fusion of Hand-crafted and Deep Features for Automatic Diabetic Foot Ulcer Classification. *TEM Journal* **2022**, *11*, 1055–1064.
97. Hüsers, J.; Hafer, G.; Heggemann, J.; Wiemeyer, S.; Przysucha, M.; Dissemond, J.; Moelleken, M.; Erfurt-Berge, C.; Hübner, U.H. Automatic Classification of Diabetic Foot Ulcer Images: A Transfer-Learning Approach to Detect Wound Maceration. In Proceedings of the Informatics and Technology in Clinical Care and Public Health. IOS Press, 2022, pp. 301–304.
98. Santos, E.; Santos, F.; Dallyson, J.; Aires, K.; Tavares, J.M.R.; Veras, R. Diabetic Foot Ulcers Classification using a fine-tuned CNNs Ensemble. In Proceedings of the International Symposium on Computer-Based Medical Systems (CBMS). IEEE, 2022, pp. 282–287.
99. Yogapriya, J.; Chandran, V.; Sumithra, M.; Elakkiya, B.; Shamila Ebenezer, A.; Suresh Gnana Dhas, C. Automated Detection of Infection in Diabetic Foot Ulcer Images Using Convolutional Neural Network. *Journal of Healthcare Engineering* **2022**, *2022*.
100. Das, S.K.; Roy, P.; Mishra, A.K. Recognition of ischaemia and infection in diabetic foot ulcer: a deep convolutional neural network based approach. *International Journal of Imaging Systems and Technology* **2022**, *32*, 192–208.
101. Das, S.K.; Roy, P.; Mishra, A.K. Fusion of handcrafted and deep convolutional neural network features for effective identification of diabetic foot ulcer. *Concurrency and Computation: Practice and Experience* **2022**, *34*, e6690.
102. Reyes-Luévano, J.; Guerrero-Viramontes, J.; Romo-Andrade, J.R.; Funes-Gallanzi, M. DFU_VIRNet: A novel Visible-InfraRed CNN to improve diabetic foot ulcer classification and early detection of ulcer risk zones. *Biomedical Signal Processing and Control* **2023**, *86*, 105341.
103. Nagaraju, S.; Kumar, K.V.; Rani, B.P.; Lydia, E.L.; Ishak, M.K.; Filali, I.; Karim, F.K.; Mostafa, S.M. Automated Diabetic Foot Ulcer Detection and Classification Using Deep Learning. *IEEE Access* **2023**, *11*, 127578–127588.
104. Biswas, S.; Mostafiz, R.; Paul, B.K.; Uddin, K.M.M.; Rahman, M.M.; Shariful, F. DFU_MultiNet: A deep neural network approach for detecting diabetic foot ulcers through multi-scale feature fusion using the DFU dataset. *Intelligence-Based Medicine* **2023**, *8*, 100128.
105. Toofanee, M.S.A.; Dowlut, S.; Hamroun, M.; Tamine, K.; Petit, V.; Duong, A.K.; Sauveron, D. Dfu-siam a novel diabetic foot ulcer classification with deep learning. *IEEE Access* **2023**.
106. Das, S.K.; Namasudra, S.; Sangaiah, A.K. HCNNet: hybrid convolution neural network for automatic identification of ischaemia in diabetic foot ulcer wounds. *Multimedia Systems* **2024**, *30*, 36.
107. Fadhel, M.A.; Alzubaidi, L.; Gu, Y.; Santamaría, J.; Duan, Y. Real-time diabetic foot ulcer classification based on deep learning & parallel hardware computational tools. *Multimedia Tools and Applications* **2024**, pp. 1–26.
108. Patel, Y.; Shah, T.; Dhar, M.K.; Zhang, T.; Niezgoda, J.; Gopalakrishnan, S.; Yu, Z. Integrated image and location analysis for wound classification: a deep learning approach. *Scientific Reports* **2024**, *14*, 7043. https://doi.org/https://doi.org/10.1038/s41598-024-56626-w.
109. Almufadi, N.F.; Alhasson, H.F.; Alharbi, S.S. E-DFu-Net: An efficient deep convolutional neural network models for diabetic foot ulcer classification. *Biomolecules and Biomedicine* **2025**, *25*, 445. https://doi.org/https://doi.org/10.17305/bb.2024.11117.
110. Ajay, A.; Bisht, A.S.; Karthik, R. Dense-ShuffleGCANet: An Attention-Driven Deep Learning Approach for Diabetic Foot Ulcer Classification Using Refined Spatio-Dimensional Features. *IEEE ACCESS* **2025**, *13*, 5507–5521. https://doi.org/https://doi.org/10.1109/ACCESS.2024.3524549.
111. Karthik, R.; Ajay, A.; Jhalani, A.; Ballari, K.; K, S. An explainable deep learning model for diabetic foot ulcer classification using swin transformer and efficient multi-scale attention-

driven network. *Scientific Reports* **2025**, *15*, 4057. https://doi.org/https://doi.org/10.1038/s41598-025-87519-1.

112. Ullah, S.; Javed, A.; Aljasem, M.; Saudagar, A.K.J. Eff-ReLU-Net: a deep learning framework for multiclass wound classification. *BMC Medical Imaging* **2025**, *25*, 257. https://doi.org/https://doi.org/10.1186/s12880-025-01785-z.
113. Reis, S.S.; Pinto-Coelho, L.; Sousa, M.C.; Neto, M.; Silva, M.; Sequeira, M. Evaluating Skin Tone Fairness in Convolutional Neural Networks for the Classification of Diabetic Foot Ulcers. *Applied Sciences* **2025**, *15*, 8321. https://doi.org/https://doi.org/10.3390/app15158321.
114. Maurya, L.; Mirza, S. MCTFWC: a multiscale CNN-transformer fusion-based model for wound image classification. *Signal, Image and Video Processing* **2025**, *19*, 574. https://doi.org/https://doi.org/10.1007/s42452-025-06745-4.
115. Fitriah, N.; Sriani, S. Classification of Foot Wound Severity in Type 2 Diabetes Mellitus Patients Using MobileNetV2-Based Convolutional Neural Network. *Journal of Applied Informatics and Computing* **2025**, *9*, 2163–2170. https://doi.org/https://doi.org/10.30871/jaic.v9i5.11015.
116. Bansal, N.; Vidyarthi, A. Multivariate Feature-based Analysis of the Diabetic Foot Ulcers Using Machine Learning Classifiers. In Proceedings of the Proceedings of the 2024 Sixteenth International Conference on Contemporary Computing, 2024, pp. 527–534. https://doi.org/https://doi.org/10.1145/3675888.3676108.
117. Karthik, R.; Ajay, A.; Bisht, A.S. Dense-ShuffleGCANet: An Attention-Driven Deep Learning Approach for Diabetic Foot Ulcer Classification Using Refined Spatio-Dimensional Features. *IEEE Access* **2024**, *13*, 5507–5521. https://doi.org/https://doi.org/10.1109/ACCESS.2024.3524549.
118. Adam, M.; Ng, E.Y.; Oh, S.L.; Heng, M.L.; Hagiwara, Y.; Tan, J.H.; Tong, J.W.; Acharya, U.R. Automated characterization of diabetic foot using nonlinear features extracted from thermograms. *Infrared Physics & Technology* **2018**, *89*, 325–337.
119. Vardasca, R.; Vaz, L.; Magalhaes, C.; Seixas, A.; Mendes, J. Towards the diabetic foot ulcers classification with infrared thermal images. In Proceedings of the Quantitative Infrared Thermography Conference, Berlin, Germany, 2018.
120. Vardasca, R.; Marques, A.; Carvalho, R.; Gabriel, J. Thermal imaging of the foot in different forms of diabetic disease. Infrared Imaging, 2015.
121. Vardasca, R.; ASIS, F. Diabetic foot monitoring using dynamic thermography and AI classifiers. *QIRT Asia, Tokyo, Japan* **2019**, pp. 1–5.
122. Selle, J.; Prakash, K.V.; Sai, G.A.; Vinod, B.; Chellappan, K. Classification of foot thermograms using texture features and support vector machine. In Proceedings of the International Conference on Electronics and Sustainable Communication Systems (ICESC). IEEE, 2021, pp. 1445–1449.
123. Padierna, L.C.; Amador-Medina, L.F.; Murillo-Ortiz, B.O.; Villaseñor-Mora, C. Classification method of peripheral arterial disease in patients with type 2 diabetes mellitus by infrared thermography and machine learning. *Infrared Physics & Technology* **2020**, *111*, 103531.
124. Cruz-Vega, I.; Hernandez-Contreras, D.; Peregrina-Barreto, H.; Rangel-Magdaleno, J.d.J.; Ramirez-Cortes, J.M. Deep learning classification for diabetic foot thermograms. *Sensors* **2020**, *20*, 1762.
125. Jain, A. DETECTION AND CLASSIFICATION OF DIABETIC FOOT THERMOGRAMS USING DEEP LEARNING. PhD thesis, Delhi Technological University, 2022.
126. Krizhevsky, A.; Sutskever, I.; Hinton, G.E. Imagenet classification with deep convolutional neural networks. *Communications of the ACM* **2017**, *60*, 84–90.
127. Rao, Y.; He, L.; Zhu, J. A residual convolutional neural network for pan-shaprening. In Proceedings of the 2017 International Workshop on Remote Sensing with Intelligent Processing (RSIP). IEEE, 2017, pp. 1–4.
128. Targ, S.; Almeida, D.; Lyman, K. Resnet in resnet: Generalizing residual architectures. *arXiv preprint arXiv:1603.08029* **2016**.
129. Redmon, J.; Farhadi, A. YOLO9000: better, faster, stronger. In Proceedings of the Proceedings of the IEEE conference on computer vision and pattern recognition, 2017, pp. 7263–7271.
130. Redmon, J.; Farhadi, A. Yolov3: An incremental improvement. *arXiv preprint arXiv:1804.02767* **2018**.

131. Khandakar, A.; Chowdhury, M.E.; Reaz, M.B.I.; Ali, S.H.M.; Hasan, M.A.; Kiranyaz, S.; Rahman, T.; Alfkey, R.; Bakar, A.A.A.; Malik, R.A. A machine learning model for early detection of diabetic foot using thermogram images. *Computers in Biology and Medicine* **2021**, *137*, 104838.
132. Khandakar, A.; Chowdhury, M.E.; Reaz, M.B.I.; Ali, S.H.M.; Abbas, T.O.; Alam, T.; Ayari, M.A.; Mahbub, Z.B.; Habib, R.; Rahman, T.; et al. Thermal Change Index-Based Diabetic Foot Thermogram Image Classification Using Machine Learning Techniques. *Sensors* **2022**, *22*, 1793.
133. Khandakar, A.; Chowdhury, M.E.; Reaz, M.B.I.; Ali, S.H.M.; Kiranyaz, S.; Rahman, T.; Chowdhury, M.H.; Ayari, M.A.; Alfkey, R.; Bakar, A.A.A.; et al. A Novel Machine Learning Approach for Severity Classification of Diabetic Foot Complications Using Thermogram Images. *Sensors* **2022**, *22*, 4249.
134. Filipe, V.; Teixeira, P.; Teixeira, A. Automatic Classification of Foot Thermograms Using Machine Learning Techniques. *Algorithms* **2022**, *15*, 236.
135. Munadi, K.; Saddami, K.; Oktiana, M.; Roslidar, R.; Muchtar, K.; Melinda, M.; Muharar, R.; Syukri, M.; Abidin, T.F.; Arnia, F. A Deep Learning Method for Early Detection of Diabetic Foot Using Decision Fusion and Thermal Images. *Applied Sciences* **2022**, *12*, 7524.
136. Anaya-Isaza, A.; Zequera-Diaz, M. Fourier transform-based data augmentation in deep learning for diabetic foot thermograph classification. *Biocybernetics and Biomedical Engineering* **2022**, *42*, 437–452.
137. Khosa, I.; Raza, A.; Anjum, M.; Ahmad, W.; Shahab, S. Automatic Diabetic Foot Ulcer Recognition Using Multi-Level Thermographic Image Data. *Diagnostics* **2023**, *13*, 2637.
138. Reyes-Luévano, J.; Guerrero-Viramontes, J.; Romo-Andrade, J.R.; Funes-Gallanzi, M. DFU_VIRnet: A Novel Visible-Infrared CNN to Improve Diabetic Foot Ulcer Classification and Early Detection of Ulcer Risk Zones. *SSRN Electron*.
139. Wannous, H.; Lucas, Y.; Treuillet, S. Enhanced assessment of the wound-healing process by accurate multiview tissue classification. *IEEE Transactions on Medical Imaging* **2010**, *30*, 315–326.
140. Mukherjee, R.; Manohar, D.D.; Das, D.K.; Achar, A.; Mitra, A.; Chakraborty, C. Automated tissue classification framework for reproducible chronic wound assessment. *BioMed Research International* **2014**, *2014*.
141. Babu, K.; Sabut, S.; Nithya, D. Efficient detection and classification of diabetic foot ulcer tissue using PSO technique. *international journal of engineering & technology* **2018**, *7*, 1006–1010.
142. Godeiro, V.; Neto, J.S.; Carvalho, B.; Santana, B.; Ferraz, J.; Gama, R. Chronic wound tissue classification using convolutional networks and color space reduction. In Proceedings of the International Workshop on Machine Learning for Signal Processing (MLSP), 2018, pp. 1–6.
143. Wijesinghe, I.; Gamage, C.; Perera, I.; Chitraranjan, C. A smart telemedicine system with deep learning to manage diabetic retinopathy and foot ulcers. In Proceedings of the Moratuwa Engineering Research Conference (MERCon), 2019, pp. 686–691.
144. Zhou, G.X.; Tao, Y.K.; Hou, J.Z.; Zhu, H.J.; Xiao, L.; Zhao, N.; Wang, X.W.; Du, B.L.; Zhang, D. Construction and validation of a deep learning-based diagnostic model for segmentation and classification of diabetic foot. *Frontiers in Endocrinology* **2025**, *16*, 1543192. https://doi.org/https://doi.org/10.3389/fendo.2025.1543192.
145. Sudarvizhi, M.D.; Nivetha, M.; Priyadharshini, P.; Swetha, J. Identification and analysis of foot ulceration using load cell technique. *IRJET* **2019**, *6*, 7792–7797.
146. Muro-De-La-Herran, A.; Garcia-Zapirain, B.; Mendez-Zorrilla, A. Gait analysis methods: An overview of wearable and non-wearable systems, highlighting clinical applications. *Sensors* **2014**, *14*, 3362–3394.
147. Maldonado, H.; Bayareh, R.; Torres, I.; Vera, A.; Gutiérrez, J.; Leija, L. Automatic detection of risk zones in diabetic foot soles by processing thermographic images taken in an uncontrolled environment. *Infrared Physics & Technology* **2020**, *105*, 103187.
148. Yap, M.H.; Ng, C.C.; Chatwin, K.; Abbott, C.A.; Bowling, F.L.; Boulton, A.J.; Reeves, N.D. Computer vision algorithms in the detection of diabetic foot ulceration: A new paradigm for diabetic foot care? *Journal of diabetes science and technology* **2016**, *10*, 612–613.
149. Yap, M.H.; Chatwin, K.E.; Ng, C.C.; Abbott, C.A.; Bowling, F.L.; Rajbhandari, S.; Boulton, A.J.; Reeves, N.D. A new mobile application for standardizing diabetic foot images. *Journal of Diabetes Science and Technology* **2018**, *12*, 169–173.

150. Yap, M.H.; Reeves, N.; Boulton, A.; Rajbhandari, S.; Armstrong, D.; Maiya, A.G.; Najafi, B.; Frank, E.; Wu, J. Diabetic foot ulcers grand challenge 2020. *DOI: https://doi. org/10.5281/zenodo* **2020**, *3715020*.
151. Cassidy, B.; Kendrick, C.; Reeves, N.D.; Pappachan, J.M.; O'Shea, C.; Armstrong, D.G.; Yap, M.H. Diabetic foot ulcer grand challenge 2021: evaluation and summary. In Proceedings of the Diabetic Foot Ulcers Grand Challenge, 2021, pp. 90–105.
152. Yap, M.H.; Kendrick, C.; Reeves, N.D.; Goyal, M.; Pappachan, J.M.; Cassidy, B. Development of Diabetic Foot Ulcer Datasets: An Overview. *Diabetic Foot Ulcers Grand Challenge* **2021**, pp. 1–18.
153. Bouallal, D.; Bougrine, A.; Harba, R.; Canals, R.; Douzi, H.; Vilcahuaman, L.; Arbanil, H. STANDUP database of plantar foot thermal and RGB images for early ulcer detection. *Open Research Europe* **2022**, *2*, 77.
154. Thomas, S., 2014.
155. Hernandez-Contreras, D.A.; Peregrina-Barreto, H.; Rangel-Magdaleno, J.D.J.; Orihuela-Espina, F. Statistical approximation of plantar temperature distribution on diabetic subjects based on beta mixture model. *IEEE Access* **2019**, *7*, 28383–28391.
156. Lucas, Y.; Treuillet, S.; Albouy, B.; Wannous, H.; Pichaud, J. 3D and color wound assessment using a simple digital camera. In Proceedings of the 9th Meeting of the European Pressure Ulcer Advisory Panel, Berlin, 2006.
157. Kręcichwost, M.; Czajkowska, J.; Wijata, A.; Juszczyk, J.; Pyciński, B.; Biesok, M.; Rudzki, M.; Majewski, J.; Kostecki, J.; Pietka, E. Chronic wounds multimodal image database. *Computerized Medical Imaging and Graphics* **2021**, *88*, 101844.
