# Supplementary material for: Advances in Image-Based Diagnosis of Diabetic Foot Ulcers Using Deep Learning and Machine Learning: A Systematic Review
Source: Biomedicines. 2025 Nov 28;13(12):2928. doi: 10.3390/biomedicines13122928 (PMC12730623; doi:10.3390/biomedicines13122928)
Supplement: Supplementary file 1 [file biomedicines-13-02928-s001.zip › S7_INPLASY Protocol 4173-1.pdf]

INPLASY PROTOCOL

To cite: Alhasson et al. New Trends in image-based Diabetic Foot Ucler Diagnosis Using Machine Learning Approaches: A Systematic Review. Inplasy protocol 2022110128. doi: 10.37766/inplasy2022.11.0128

Received: 25 November 2022

Published: 25 November 2022

Corresponding author:
Haifa F. Alhasson

hhson@qu.edu.sa

Author Affiliation:
Department of Information Technology, College of Computer, Qassim University, Buraydah, Saudi Arabia.

Support: **Deputyship for Research& Innovation, Ministry of Education, and Qassim University, Saudi Arabia.**

Review Stage at time of this submission: **Completed but not published.**

Conflicts of interest:
None declared.

Advances in Image-Based Diagnosis of Diabetic Foot Ulcers Using Deep Learning and Machine Learning: A Systematic Review

Alhasson, HF[1]; Alharbi, SS[2].

Review question / Objective: A significant amount of research has been conducted to detect and recognize diabetic foot ulcers (DFUs) using computer vision methods, but there are still a number of challenges. DFUs detection frameworks based on machine learning/deep learning lack systematic reviews. With Machine Learning (ML) and Deep learning (DL), you can improve care for individuals at risk for DFUs, identify and synthesize evidence about its use in interventional care and management of DFUs, and suggest future research directions.
Information sources: A thorough search of electronic databases such as Science Direct, PubMed (MIDLINE), arXiv.org, MDPI, Nature, Google Scholar, Scopus and Wiley Online Library was conducted to identify and select the literature for this study (January 2010-January 01, 2023). It was based on the most popular image-based diagnosis targets in DFu such as segmentation, detection and classification. Various keywords were used during the identification process, including artificial intelligence in DFu, deep learning, machine learning, ANNs, CNNs, DFu detection, DFu segmentation, DFu classification, and computer-aided diagnosis.

INPLASY registration number: This protocol was registered with the International Platform of Registered Systematic Review and Meta-Analysis Protocols (INPLASY) on 25 November 2022 and was last updated on 4 November 2025 (registration number INPLASY2022110128).

INTRODUCTION

Review question / Objective: A significant amount of research has been conducted to detect and recognize diabetic foot ulcers (DFUs) using computer vision methods, but there are still a number of challenges. DFUs detection frameworks based on machine learning/deep learning lack systematic reviews. With Machine Learning

Alhasson et al. Inplasy protocol 2022110128. doi:10.37766/inplasy2022.11.0128 Downloaded from https://inplasy.com/inplasy-2022-11-0128/

(ML) and Deep learning (DL), you can improve care for individuals at risk for DFUs, identify and synthesize evidence about its use in interventional care and management of DFUs, and suggest future research directions.

Condition being studied: Recognize diabetic foot ulcers (DFUs).

METHODS

Search strategy: Full-length articles were retrieved from the journals. As part of the screening process, the two authors organize a focus group in order to ensure that the eligibility criteria and inclusion criteria are met. A list of the titles, authors, dates of publication, places of publication, and full abstracts of the literature obtained through the above-mentioned search protocol was imported into Microsoft Excel. Using the software, duplicates were removed from the list of literature and remaining article abstracts were screened using eligibility criteria.
The required articles for this review study were selected in two stages. The first stage was the selection of articles based on the title and abstracts related to our research topic. The preliminary search yielded 5228 articles that were appropriate to address the study's aim, then due to duplication, 4012 articles were removed. Hence, the two authors retrieved 1216 articles at the second stage of selection. In the next stage, they followed a criterion to include research papers. For the purposes of the review, all authors were satisfied with the exclusion and inclusion of papers. In order to avoid missing relevant literature, criteria were devised after a focus group consisting of the two authors above reviewed preliminary papers.

Participant or population: N/A.

Intervention: N/A.

Comparator: N/A.

Study designs to be included: This review was conducted in accordance with PRISMA guidelines for preferred reporting items for systematic reviews and meta-analyses of diagnostic test accuracy studies.

Eligibility criteria: The article must be focused on AI, and its application should be one of the related assigned dentistry applications and including the statistical analysis for the results. The article must include reference to or creation of datasets that are used to assess a model. The articles that full text.

Information sources: A thorough search of electronic databases such as Science Direct, PubMed (MIDLINE), arXiv.org, MDPI, Nature, Google Scholar, Scopus and Wiley Online Library was conducted to identify and select the literature for this study (January 2010-January 01, 2023). It was based on the most popular image-based diagnosis targets in DFu such as segmentation, detection and classification. Various keywords were used during the identification process, including artificial intelligence in DFu, deep learning, machine learning, ANNs, CNNs, DFu detection, DFu segmentation, DFu classification, and computer-aided diagnosis.

Main outcome(s): The most representative articles covering the area of diabetic foot detection based on machine learning, published in journals and impact conferences, were investigated between 2015 and 2021, focusing on the interval 2018–2022 as new trends.Additionally presented are the main databases and trends in their use in training diabetic foot detection models. Finally, a research agenda was highlighted to advance the field towards the new trends.

Quality assessment / Risk of bias analysis: A variety of specific areas of the DFUs have been assessed for AI's diagnostic accuracy throughout the studies. In order to assess the risk of bias, QUADAS-2a commonly used tool in the literature, was used.

Strategy of data synthesis: In order to have a reasonable comparison ,it is important to compare the analysed papers based on their common statistical performances

Alhasson et al. Inplasy protocol 2022110128. doi:10.37766/inplasy2022.11.0128 Downloaded from https://inplasy.com/inplasy-2022-11-0128/

metrics. The performance evaluation metrics most used in SL detection, segmentation, and classification are the following: Accuracy, Precision, Sensitivity, Specificity, F1-score, and Jaccardindex.

Subgroup analysis: N/A.

Sensitivity analysis: N/A.

Country(ies) involved: Saudi Arabia.

Keywords: Diabetes Mellitus; Diabetic foot ulcers (DFu); DFU dataset; Machine Learning (ML);Deep Learning (DL); Convolutional Neural Network (CNN);Thermogram.

Contributions of each author:

Author 1 - Haifa F. Alhasson.
Email: hhson@qu.edu.sa
Author 2 - Shuaa S. Alharbi.
Email: shuaa.s.alharbi@qu.edu.sa

Alhasson et al. Inplasy protocol 2022110128. doi:10.37766/inplasy2022.11.0128 Downloaded from https://inplasy.com/inplasy-2022-11-0128/
